# Bone-derived factors mediate crosstalk between skeletal and extra-skeletal organs

**DOI:** 10.1038/s41413-025-00424-1

**Published:** 2025-04-30

**Authors:** Tailin He, Lei Qin, Sheng Chen, Shaochuan Huo, Jie Li, Fuping Zhang, Weihong Yi, Yifang Mei, Guozhi Xiao

**Affiliations:** 1 Department of Rheumatology and Immunology, Shenzhen Third People’s Hospital, Shenzhen, 518112 China; 2 CAS Key Laboratory of Pathogenic Microbiology and Immunology, Institute of Microbiology, Chinese Academy of Sciences (CAS), 100101 Beijing, China; 3 Department of Biochemistry, Homeostatic Medicine Institute, School of Medicine, Shenzhen Key Laboratory of Cell Microenvironment, Guangdong Provincial Key Laboratory of Cell Microenvironment and Disease Research, Southern University of Science and Technology, Shenzhen, 518055 China; 4 Department of Orthopedics, Shenzhen Nanshan People’s Hospital, and the 6th Affiliated Hospital of Shenzhen University Medical School, Shenzhen, 518052 China; 5 Department of Orthopaedics, Union Hospital, Tongji Medical College, Huazhong University of Science and Technology, Wuhan, 430022 China; 6 Shenzhen Hospital of Guangzhou University of Chinese Medicine (Futian), Shenzhen 518000, China, Shenzhen Research Institute of Guangzhou University of Traditional Medicine (Futian), Shenzhen, 518000 China

**Keywords:** Bone, Osteoporosis, Osteogenesis imperfecta

## Abstract

Bone has long been acknowledged as a fundamental structural entity that provides support and protection to the body’s organs. However, emerging research indicates that bone plays a crucial role in the regulation of systemic metabolism. This is achieved through the secretion of a variety of hormones, cytokines, metal ions, extracellular vesicles, and other proteins/peptides, collectively referred to as bone-derived factors (BDFs). BDFs act as a medium through which bones can exert targeted regulatory functions upon various organs, thereby underscoring the profound and concrete implications of bone in human physiology. Nevertheless, there remains a pressing need for further investigations to elucidate the underlying mechanisms that inform the effects of bone on other body systems. This review aims to summarize the current findings related to the roles of these significant modulators across different organs and metabolic contexts by regulating critical genes and signaling pathways in vivo. It also addresses their involvement in the pathogenesis of various diseases affecting the musculoskeletal system, circulatory system, glucose and lipid metabolism, central nervous system, urinary system, and reproductive system. The insights gained from this review may contribute to the development of innovative therapeutic strategies through a focused approach to bone secretomes. Continued research into BDFs is expected to enhance our understanding of bone as a multifunctional organ with diverse regulatory roles in human health.

## Introduction

The skeletal system constitutes a specialized form of connective tissue characterized by rigidity, functioning to provide structural support while safeguarding internal organs. There are three distinct types of bone cells: osteoblasts, osteoclasts and osteocytes.^1^ In adult human bones, osteoblasts make up about 5% of total bone cells, while osteoclasts account for 1%, and the remaining 90%–95% are osteocytes.^2^ The skeleton is essential for maintaining mechanical integrity and regulating calcium and phosphorus levels. Its high remodeling activity and vascularization imply that bone tissue has broader physiological contributions to the entire body.^3^ Traditionally considered a structural organ for body movement and organ protection, recent research indicates that the skeleton can also secrete many kinds of bone-derived factors (BDFs) and contribute to the pathophysiology of many diseases.^4^

**Fig. 1:**
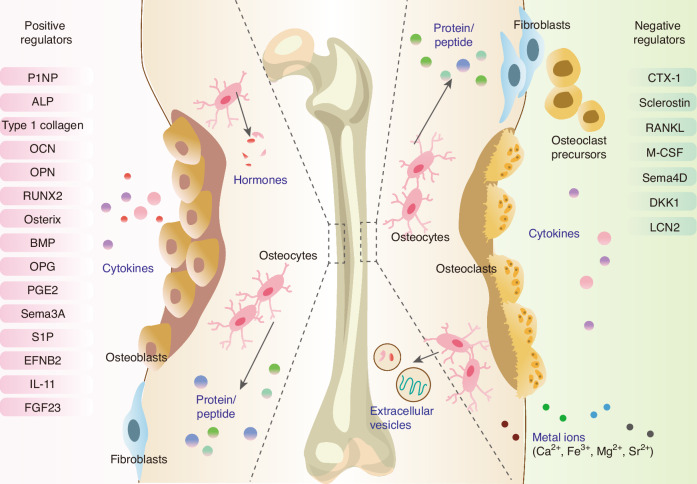
The classifications of Bone-Derived Factors (BDFs). BDFs comprise a diverse array of hormones, extracellular vesicles, cytokines, and other proteins or peptides secreted by osteoblasts, osteoclasts, and osteocytes. They play a crucial role by influencing multiple organs and tissues, including the skeletal system, musculature, liver, adipose tissue, pancreas, brain, testes, kidneys, and immune system

BDFs include various hormones, cytokines, metal ions, extracellular vesicles (EVs), and other proteins/peptides released from the skeleton (Fig. 1). The synthesis and subsequent functionality of BDFs are multifaceted processes encompassing transcription, translation, secretion, and receptor binding.^5^ BDFs play a crucial role in maintaining homeostasis within organisms by influencing various organs and tissues, including the skeletal system, muscular tissue, liver, adipose (fat) tissue, pancreas, central nervous system (CNS), testicular structures, and renal system.^6–11^ Importantly, these factors also play important roles in glucose and lipid metabolism, cholesterol regulation, and insulin sensitivity, affecting both skeletal and extra-skeletal organs.^12,13^ BDFs exhibit specific activity on distinct target organs, a process determined by the anatomical localization of their corresponding receptors.^14^ Some prospective clinical studies have highlighted the significance of BDFs as potential biomarkers for predicting, preventing, and managing various diseases.^11^ Further exploration of the function involving factors derived from bone tissue may yield innovative perspectives on therapeutic approaches for diseases. As a result, a concise review of the regulatory role of the skeleton in other body systems is necessary.

This review focuses on up-to-date perspectives regarding the origins of prevalent BDFs with the aim of understanding their fundamental biology—drawing from the discoveries of both experimental and clinical research. In this review, we aim to summarize the roles of BDFs in the musculoskeletal system, glucose and lipid metabolism, CNS, circulatory system, urinary system, and reproductive system.

## BDFs and the musculoskeletal system

**Fig. 2:**
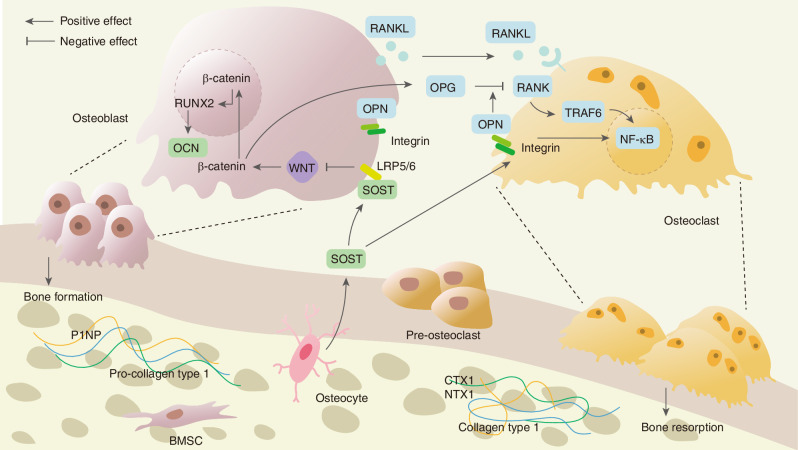
The role of BDFs in regulating bone homeostasis. Bone formation markers, including P1NP/P1CP, osteocalcin (OCN), osteopontin (OPN), and sclerostin, alongside bone resorption markers such as CTX1/NTX1 and RANKL, play a crucial role in maintaining the equilibrium of bone remodeling. This dynamic process facilitates continuous bone renewal, thereby ensuring stable bone mass and structural integrity

The interaction between BDFs and the musculoskeletal system is a complex and multifaceted process. The human skeletal system comprises two principal divisions: the axial and appendicular skeletons. The axial skeleton establishes the central body axis, including the skull, vertebral column, thoracic cage (ribs and sternum), whereas the appendicular skeleton serves as the attachment framework for extremities, composed of limb bones and their associated girdles. Bone development occurs through two fundamental mechanisms: intramembranous and endochondral ossification.^15^ Intramembranous ossification describes the process whereby mesenchymal tissue undergoes direct differentiation into osseous material, predominantly occurring in flat bones including cranial bones and the clavicle. Endochondral ossification, on the other hand, involves the formation of a bone through a cartilage intermediate, which is the method used in the formation of long bones. Long bones are composed of a dense outer layer known as cortical bone, which provides strength and support, and an inner, spongy bone called trabecular bone, which is more metabolically active and plays a crucial role in bone remodeling and mineral homeostasis. Cortical bone forms the shafts of long bones, while trabecular bone is found at the ends of long bones and in the interior of other bones. Long bones consist of a long shaft (the diaphysis) and two articular (joint) surfaces called epiphyses. The diaphysis is primarily composed of compact bone, while the epiphyses contain spongy bone and are covered with articular cartilage for smooth joint movement. Bone metabolism is a complex process that involves the continuous removal of old bone by osteoclasts and the replacement of new bone by osteoblasts regardless of age and health status.^16^ Bone resorption commences with osteoclast-mediated resorption of discrete bone volumes at anatomically defined skeletal sites, generating resorption pits (Howship’s lacunae).^17^ The resorptive phase typically spans 4–6 weeks, during which bone matrix constituents undergo enzymatic degradation and release into the bone microenvironment.^18,19^ Catabolized components are translocated into systemic circulation, with partial renal elimination through urinary excretion.^20^ Circulating levels of bone-derived elements and their catabolites serve as measurable indicators of resorptive activity.^21^ Afterwards, osteoblasts migrate to the resorption pit to commence bone formation through synthesis of osteoid matrix, a collagen-dense connective tissue framework, which becomes detectable in peripheral blood as bone formation biomarkers.^22^ This osteogenic phase culminates in matrix mineralization, completing the remodeling cycle within 4–5 months. The spatiotemporal coordination of these processes at discrete anatomical sites constitutes the coupling phenomenon, localized within functional bone remodeling units. This turnover mechanism facilitates replacement of mechanically compromised bone tissue, thereby preserving structural integrity while regulating mineral homeostasis and acid-base equilibrium. In the musculoskeletal system, muscle strength is determined by the integrity and function of muscle fibers. Bone-derived endocrine factors secreted during active remodeling phases appear mechanistically associated with musculoskeletal functional preservation. This association arises through dual mechanisms: biomechanical leverage via skeletal structural support systems and provision of anatomical attachment sites for skeletal muscle fibers, combined with endocrine regulation of muscular physiology.^23^ BDFs, such as C-terminal telopeptide (CTX), procollagen one amino-terminal propeptide (P1NP), osteocalcin, osteopontin (OPN), sclerostin, receptor activator of nuclear factor kappa B Ligand (RANKL), prostaglandin E2 (PGE2) and bone morphogenetic proteins (BMPs), play crucial roles in these processes by multiple signaling pathways (Fig. 2). Several studies have highlighted the importance of these factors in mediating the bone metabolism.

### CTX-1 and P1NP

Bone remodeling encompasses two fundamental processes: the resorption of aged bone and the synthesis of new bone. P1NP serves as a biomarker for bone formation, while CTX-1 is indicative of bone resorption, both of which are endorsed for clinical application.^17^ Bone remodeling occurs in both trabecular and cortical bone. Quantitative equilibrium exists between resorbed and neoformed bone volumes within individual remodeling units, establishing remodeling equilibrium that enables continuous osseous regeneration. Nevertheless, the amount of new bone formed at each unit is reduced compared to the resorption in the same cycle, leading to a remodeling imbalance. In individuals diagnosed with osteoporosis, the assessment of bone remodeling markers may prove beneficial in evaluating the efficacy of anabolic and antiresorptive treatments, monitoring adherence to therapeutic regimens, or identifying potential cases of secondary osteoporosis.

P1NP is a cleavage product released during the extracellular processing of type 1 collagen and is a marker of bone formation.^24^ Osteoblasts originate from mesenchymal stem cells and synthesize osteoid.^25^ Upon completion of their tasks, osteoblasts either undergo apoptosis, transition into lining cells, or become embedded in bone as osteocytes. Type I collagen, constituting 90% of total bone protein, is the most prevalent bone protein.^26^ It is initially secreted as procollagen, followed by enzymatic cleavage of the N-propeptides and C-propeptides. The deposition of type I collagen forms a quarter-stagger array interconnected by pyridinium crosslinks, specifically deoxypyridinoline and pyridinoline. Osteoblast lineage cells demonstrate phase-specific biosynthetic peaks: collagen production predominates during proliferation, bone alkaline phosphatase reaches maximal expression in the matrix maturation phase, and osteocalcin secretion peaks during mineralization.^27^ This temporal regulation of biomarker synthesis mechanistically explains the discrepant serum concentration profiles observed during osteoblast differentiation. Osteoblasts contain a substantial amount of type I procollagen. In bone formation, type I procollagen is secreted into the extracellular space and enzymatically cleaved into three fragments: P1NP, C-terminal peptide of type 1 procollagen (P1CP), and type I collagen. Type I collagen is then assembled into osteoid, where inorganic minerals (calcium and phosphorus) deposit and form hydroxyapatite (osteoid mineralization). P1NP and P1CP are released into the blood and urine as metabolites, and detecting their levels can reflect the extent of bone formation. Due to the considerable research evidence on P1NP’s reflection of bone formation compared to P1CP, P1NP is considered a superior biomarker for bone formation. It exhibits a high specificity, is minimally influenced by circadian rhythms and food intake, and remains stable at room temperature. Presently, P1NP is widely recommended internationally as the preferred biomarker for bone formation. Serum P1NP is mainly derived from bone, exhibits mild circadian fluctuations, and rises during therapies that stimulate bone formation. It also has been shown to have a strong association with the extent of bone metastases in patients with advanced prostate cancer.^28^ The mechanisms underlying the regulatory effects of CTX-1 and P1NP on bone metabolism involve complex signaling networks that remain unclear. Understanding these regulatory mechanisms is crucial for developing effective strategies to manipulate bone metabolism and treat bone diseases such as osteoporosis.

Osteoclasts originate from cells within the monocyte-macrophage lineage.^29^ Post-resorption, osteoclasts initiate programmed cell death. These bone-resorbing cells establish mineral dissolution through sealing zone-mediated bone attachment and acid secretion. The low-pH microenvironment activates osteoclast-derived proteolytic enzymes including cathepsin K, which degrade collagenous matrices to release type I collagen fragments such as NTX-1 and CTX-1. Clinically validated assays quantify these biomarkers in biological specimens (serum, plasma, urine). TRAP5b (ACP5), an osteoclast-specific isoenzyme of tartrate-resistant acid phosphatase, reflecting the number of osteoclasts.^30^ It differs from NTX-1 and CTX-1, which indicate osteoclast activity. CTX-1 is a degradation product of type I collagen, which is the main component of bone matrix and contributes to accelerated bone loss and osteoporosis.^31^ It is released into the circulation during bone resorption and serves as a marker of bone turnover. Elevated serum CTX-1 concentrations in postmenopausal H-type hypertension patients demonstrate osteoclastic activation potentiation, driving upregulated inflammatory mediator production that exacerbates osteoporosis progression.^32^ However, a recent systematic review indicates that CTX-1 quantification lacks predictive validity for bisphosphonate-treated patients’ osteonecrosis risk stratification.^33^ There is a strong interconnection between bones and muscles, both mechanically and biochemically. Hormones released during the process of bone remodeling could potentially influence the preservation of muscle mass and strength. Several randomized controlled trials have suggested that the levels of CTX-1 and P1NP, as biomarkers, are correlated with muscle functionality, particularly in the elderly population.^34,35^

### Osteocalcin

As a mineralized framework, bone serves to shield internal organs from potential harm that could lead to fatality in the event of injury. Bone formation is crucial for the longitudinal expansion of the skeletal structure, while bone remodeling was initially intended to mend small and large-scale damages, such as fractures. The OCN protein contains 49 amino acids in humans and 46 amino acids in rodents, which generate a small protein with a molecular weight of 5 kD in bone matrix.^13^ Regardless of its small size, OCN is the most abundant non-collagenous protein in bone, as well as the 10^th^ most abundant protein in the body.^36^ OCN is primarily synthesized by mature osteoblasts and plays a crucial role in a variety of regulatory functions, including bone formation, mineralization, and muscle mass.^13^ Generally, OCN has two different forms, carboxylated osteocalcin (cOCN) and undercarboxylated osteocalcin (ucOCN) in bodies. Once OCN proteins are generated, a post-translational modification, carboxylation, is operated by the enzyme gamma carboxylase at three glutamate residues (17, 21 and 24 in humans) in the presence of vitamin K as a co-factor in cells.^36^ This carboxylation confers OCN into cOCN with a high affinity for calcium and phosphate ions and mineral structures in bone. This cOCN is the first form of OCN, which is the most abundant non-collagenous protein and regulates bone ECM mineralization.^13^ The acidic microenvironment of bone resorption induces ucOCN biosynthesis, which enters systemic circulation as the endocrine-active isoform of osteocalcin. This decarboxylated protein exerts dual regulatory functions: suppressing physiological hydroxyapatite crystallization through crystal surface interactions, and attenuating bone matrix mineralization capacity. Furthermore, UcOCN modulates mineral deposition kinetics in calcified tissues via nucleation suppression mechanisms, and its local concentration is linked to pathological calcification, where higher levels induce increased deposition of hydroxyapatite in various calcified tissues.^37^ Conversely, mice deficient in OCN exhibit elevated bone volume with preserved microarchitectural integrity, manifesting as cortical thickening and volumetric density augmentation.^38^ Osteocalcin-null rat models also display enhanced biomechanics and increased trabecular bone, as evidenced by a persistent increase in failure load in osteocalcin-deficient rats with or without ovariectomy.^39,40^ Paradoxical to its classical characterization as a bone matrix mineralization suppressor, osteocalcin upregulation demonstrates biomechanical competence augmentation under mechanical loading conditions. Mechanical stimulation (e.g., physical training) induces osteocalcin elevation, concomitantly enhancing musculoskeletal performance and lean tissue accrual.^41^ Exogenous osteocalcin administration improves bone biomechanical properties, positioning this molecule as a therapeutic candidate for reducing fracture susceptibility associated with bone fragility syndromes.

When male mice lacking the *Ocn* gene in the 129Sv genetic background were bred with wild-type female mice, the litter size was smaller compared to wild-type counterparts.^42^ Additionally, there was a decrease in the frequency of litters. Furthermore, the *Ocn*^−/−^ mouse line generated by Karsenty’s group exhibited reduced muscle mass.^42^ It is demonstrated that osteocalcin signaling in myofibers is essential for maintaining muscle mass in mice, partly through promoting protein synthesis in myotubes without affecting protein breakdown. Moreover, it was shown that treatment with exogenous Ocn for 28 days was adequate to increase muscle mass in 9-month-old wild-type mice. Ocn is both necessary and sufficient to prevent age-related muscle loss in mice.

Mice possess an Ocn gene cluster, consisting of *Bglap*, *Bglap2* and *Bglap3* within a 23-kb span of genomic DNA. In contrast, humans and rats have a single OCN gene (*BGLAP*).^43,44^ The *Bglap* and *Bglap2* genes exhibit osteoblast-restricted transcriptional activity, whereas *Bglap3* demonstrates ectopic expression in non-osseous anatomical sites (renal parenchyma, pulmonary tissue, male reproductive organs). Osteocalcin transcriptional regulation is governed by the runt-related transcription factor 2 (RUNX2), a master transcriptional regulator of osteoblastic lineage commitment.^45^ Genetic ablation of *Runx2* in murine models results in complete Ocn ablation, corroborated by antisense oligonucleotide-mediated dose-dependent suppression in rat primary osteoblasts and ROS17/2.8 osteosarcoma cells. Complementary gain-of-function studies revealed Runx2-mediated ectopic Ocn activation in multipotent mesenchymal progenitor populations. Therefore, the regulatory mechanism of OCN genes differs among mice, humans, and rats. The specific activities of Bglap and Bglap2 in osteoblasts, along with the expression pattern of Bglap3 in non-osteoid tissues, underscore the complex regulation of OCN gene expression. These findings enhance our understanding of the molecular pathways involved in bone development and metabolism across different species.

In addition to osteoblasts, circulating osteogenic precursor (COP) cells express surface markers associated with bone formation, including OCN, alkaline phosphatase, and type 1 collagen.^46^ The prevalence of COP cells in healthy individuals remains relatively low and stable, with an increase observed in response to factors such as fractures, thyroid hormones, and hypoxia. These cells are drawn to the fracture site by stromal cell-derived factor-1, which is expressed during osteogenesis, facilitating their migration to the site of injury to aid in bone tissue repair.

### Osteopontin

Osteopontin (OPN) is a secreted phosphoprotein that is abundant in bone and has been implicated in a variety of physiological and pathological processes, including bone metabolism. OPN is produced by osteoblasts, osteoclasts, and other cells in the bone microenvironment and plays a crucial role in bone remodeling and skeletal development.^47^ OPN regulates bone formation by modulating osteoblast function. OPN stimulates osteoblast differentiation and bone formation by interacting with integrins on the surface of osteoblasts.^47^ OPN also promotes mineralization by inducing the expression of alkaline phosphatase and OCN, which are critical for bone mineralization. BMP is a member of the transforming growth factor-β superfamily and plays a crucial role in bone development and repair.^48^ BMPs regulate bone formation by promoting osteoblast differentiation. BMPs bind to receptors on osteoblast precursors, stimulating the expression of osteogenic transcription factors, such as Runx2 and Osterix.^48^ BMPs also promote mineralization by inducing the expression of OPN and osteonectin, which are involved in the mineralization of the extracellular matrix.^49^ BMPs downregulate the expression of RANKL, a key factor for osteoclastogenesis, leading to a reduction in osteoclast formation and activity.^50^ In addition, OPN is overexpressed in skeletal muscle injuries that occur due to sports or trauma. A specific genetic variation in the promoter region of the *OPN* gene, known as rs28357094, has been linked to various inflammatory conditions and muscle mass in healthy, young individuals.^51^ OPN appears to play a role in regulating both muscle generation and inflammation during the initial phase of muscle tissue repair.^52,53^ It can enhance muscle healing after injury by activating matrix metalloproteinase (MMP) and TGF-β pathways.^54^

### Sclerostin

Sclerostin is a glycoprotein encoded by the *SOST* gene that is characterized by a cysteine-knot structure, predominantly secreted by osteocytes in the bone tissue. This 24 kDa protein is a suppressor for the bone formation process by inhibiting canonical Wnt and BMP signaling pathway, exerting a negative regulatory influence on bone formation and mineralization.^55^ Sclerostin, upon secretion, binds to the low-density lipoprotein receptor-related protein-4 (LRP4) receptor on the osteoblast membrane, thereby being sequestered within the bone cavity.^56^ Sclerostin can also bind to LRP5/6 receptors on osteoblasts and osteocytes, antagonizing the Wnt signaling pathway, which is essential for bone formation and bone mass regulation.^57^ When sclerostin binds to LRP5/6 receptors, it prevents the recruitment of co-receptors essential for Wnt signaling transduction, leading to the degradation of β-catenin and the inhibition of osteoblastogenesis.^58^ Sclerostin downregulates the expression of alkaline phosphatase, a key enzyme involved in mineralization, leading to a reduction in bone mineralization. This effect is achieved through the modulation of RUNX2 and Osterix expression, transcription factors that regulate osteoblast differentiation and mineralization. In addition to its direct effects on osteoblasts, sclerostin also modulates bone metabolism through antagonizing the effects of BMPs on osteoblast differentiation and bone formation.^59^ This interaction suggests a potential mechanism for maintaining bone homeostasis through the crosstalk between different BDFs.

The negative regulation of bone mass of sclerostin has made it a promising target for anti-osteoporosis drugs. Due to its wonderful performance in both enhancing bone formation and reducing bone resorption, Romosozumab, a human monoclonal antibody directly against sclerostin, was recently approved in several countries for the treatment of severe osteoporosis in postmenopausal women with high fracture risk.^60–62^ However, as clinical trials progress, some studies indicate that sclerostin inhibition may lead to hypertension, type 2 diabetes, myocardial infarction, and coronary artery calcification, thereby increasing cardiovascular disease risk.^63^ This side effect has been included in Romozumab’s ‘black box warning’, highlighting the potential for serious, life-threatening adverse reactions, possibly due to sclerostin’s protective role in vasculature. Systemic sclerostin inhibition promotes vascular calcification and elevates inflammatory cytokine levels, further heightening cardiovascular disease risk.^64^ Consequently, bone-targeted Sost inhibition warrants careful consideration in treatment strategies. For instance, Gao et al.^65^ recently found that Sost knockout in bone marrow Adipoq^+^ cells activates the Wnt signaling pathway in mesenchymal stromal cells, enhancing osteogenic differentiation and significantly increasing bone mass in mice without notably affecting sclerostin or adipose tissue markers in peripheral circulation. This observation may inspire novel targeted therapies for osteoporosis.

Focal adhesion proteins play a pivotal role in chondrogenesis and the initial stages of skeletal development through sclerostin. Conditional knockout of Kindlin-2 in osteocytes and terminally differentiated osteoblasts provoked coordinated cellular shifts within the osseous niche: osteoblast lineage cell depletion concurrent with osteoclast-adipocyte lineage expansion.^66^ Mechanistically, Kindlin-2 ablation induced sclerostin hyperexpression in osteocytes coupled with β-catenin suppression in osteoblasts, concomitantly suppressing osteoblastogenesis and lineage commitment.^67^ Osteocytes deficient in Pinch and the bone slice cultures derived from Pinch knockout mice exhibited high levels of sclerostin, which significantly inhibited the differentiation of osteoblasts in primary BMSCs and calvarial cultures.^68^

Muscles, akin to bones, have been identified as endocrine organs due to their ability to produce and release hormone-like substances that can influence each other and other tissues, leading to a concept known as “bone–muscle crosstalk“.^69^ Recent research has suggested a potential role for sclerostin in myogenesis, thereby modulating the interaction between bone and muscle.^70^ Sclerostin inhibits the Wnt signaling pathway, which is known to have bone-protective effects by decreasing the production of receptor activators of RANK-L, increasing the production of OPG, and promoting the transcription of genes essential for osteoblast survival and activity. Additionally, the Wnt pathway positively impacts skeletal muscle by enhancing myoblast differentiation and reducing the quiescence of satellite cells, which in turn improves muscle mass and function. Consequently, targeting the Wnt pathway through the inhibition of sclerostin could potentially combat osteosarcopenia and its associated clinical implications.^70,71^

### RANKL/RANK/OPG system

RANKL/RANK/OPG is a triad of the ligand/signaling receptor/decoy receptor that plays pivotal roles in bone/cartilage metabolism and the immune system. In this triad, the receptor activator of nuclear factor kappa B ligand (RANKL) serves as a ligand required for osteoclast generation, and RANK serves as the receptor for RANKL, and osteoprotegerin (OPG) acts as a decoy receptor for RANKL. RANK was first discovered by Andreson et al. as a member of the tumor necrosis factor receptor (TNFR) family.^72^ This research group also isolated RANKL as a RANK ligand by direct expression screening in the dendritic cells, which stimulates naive T-cell proliferation and survival by interacting with RANK in T cells.^72^ Concurrently, Dougall et al.^73^ provided evidence of RANK as the in vivo receptor for RANKL by illustrating similar phenotypes of RANK knockout mice and RANKL knockout mice. OPG was discovered by Simonet and colleagues in 1997 as a novel secreted protein involved in bone density regulation.^74,75^

Molecularly, RANK is a type I transmembrane glycoprotein and mainly expressed in osteoclast precursors, mature osteoclasts, epithelial cells, hypertrophic chondrocytes, and immune cells such as DCs, macrophages, and microglia.^76–79^ RANKL-RANK ligation triggers the recruitment of intracellular signaling mediators through TRAF2/5/6 adapter molecules, activating modular assembly of NF-κB, c-Jun N-terminal kinase (JNK), and Src signaling pathways.^80^ This molecular cascade culminates in transcriptional reprogramming required for osteoclast differentiation. RANKL regulates osteoclastogenesis, osteoclast differentiation, bone–cartilage crosstalk, and function during bone remodeling.^79,81^ RANKL has three isoforms coded from a single gene by alternative splicing in humans, i.e., two of these isoforms are type II transmembrane-bound glycoproteins and the third isoform is a soluble ligand (sRANKL) which lacks both the transmembrane and cytoplasmic domains.^82^ sRANKL is also generated by the matrix-metalloproteinase 14 (MMP14), which directly cleaves the ectodomain of the membrane-anchored RANKL and transforms it into a soluble form.^83^ These published data suggest that the membrane-anchored RANKL isoform facilitates juxtacrine signaling through direct osteoclast-precursor cellular interactions, whereas sRANKL undergoes paracrine dissemination enabling systemic activation of responsive cell populations.^80^ The loss of Kindlin-2 resulted in elevated RANKL expression in osteocytes, subsequently enhancing osteoclastogenesis and bone resorption.^66^ The ablation of Kindlin-2 in osteocytes facilitates osteoclast formation in cocultures of osteocytes and bone marrow monocytes, a process that can be significantly inhibited by an anti-RANKL-neutralizing antibody. Additionally, Pinch deficiency upregulates sclerostin and RANKL expression in hypertrophic zone chondrocytes, thereby establishing a dual regulatory mechanism: coordinated suppression of osteogenesis coupled with enhanced osteolytic activity, culminating in bone mass depletion.^68,84^ The human monoclonal antibody Denosumab (anti-RANKL neutralizing agent) has attained therapeutic approval for managing osteoporosis and malignancy-associated skeletal pathologies, demonstrating targeted RANKL inhibition efficacy across international clinical practice settings.^85^

OPG functions as a soluble decoy receptor through high-affinity RANKL binding, thereby competitively inhibiting RANK-mediated ligand-receptor interaction, which further inhibits osteoclast differentiation, activation, and survival. OPG also belongs to the TNF receptor superfamily. Hsu and colleagues^86^ developed transgenic mice overexpressing RANK and OPG, both of which showed osteopetrosis. Moreover, mice deficient with the *Rank* gene (*Rank*^*−/−*^) displayed smaller body size, and shortened limbs with enhanced endosteal mineralization within bone marrow compartments relative to wild-type counterparts.^73^ These mice also showed inhibited osteoclast differentiation and maturation. Furthermore, mice with a disrupted *Rankl* gene (*Rankl*^*−/−*^) also show severe osteopetrosis and a defect in tooth eruption and completely lack osteoclasts.^76^ However, deletion of the *Opg* gene in mice (*Opg*^*−/−*^) caused severe osteoporosis with decreased BMD and high bone turnover rate, disorganized matrix, and increased mortality compared with their control littermates.^87,88^

Together, these results suggest that the RANKL/RANK/OPG system is essential for bone microenvironment.

### Prostaglandin E2

The hypothalamus plays a crucial role in maintaining whole-body homeostasis by synthesizing peripheral information and orchestrating the activity of various peripheral organs via descending neural or neuroendocrine pathways.^89^ The hypothalamic regulation of skeletal homeostasis involves mechanoresponsive detection of skeletal prostaglandin E2 (PGE2) concentrations. Functioning as a potent osteoanabolic mediator, PGE2 exhibits load-dependent accumulation in mechanically stimulated osseous tissue, with exogenous administration directly stimulating osteogenesis.^90^ Pharmacological suppression of prostaglandin biosynthesis compromises bone mechanosensing capacity. Mechanistically, fluid shear stress stimulation in osteoblasts upregulates cyclooxygenase-2 (COX2) expression, the rate-limiting enzyme for PGE2 production. Skeletal interoceptive regulation of bone homeostasis has been mechanistically linked to PGE2-mediated activation of intraosseous sensory neurons, initiating a neuroendocrine cascade characterized by hypothalamic CREB phosphorylation (ascending signaling) and subsequent downregulation of sympathetic tone governing osteoblast activity (descending modulation).^91^ This interoceptive axis further enables biomaterial-driven osteogenesis potentiated by divalent metal cations that augment macrophage-derived PGE2 secretion.^92^ Therefore, PGE2 demonstrates mechanotransduction capabilities by mediating biomechanical-biochemical signal integration through skeletal somatosensory networks. Quantitatively, human skeletal musculature exhibits fiber-type-specific disparity in both PGE2 biosynthetic capacity and cognate receptor density, with distinct expression profiles observed between oxidative (type I) and glycolytic (type II) fiber subtypes. With aging, there is an increase in PGE2 production capacity, while receptor levels are reduced in skeletal muscle. These observations suggest that the PGE2/CO2 signaling axis and its pharmacologic modulators orchestrate molecular adaptation processes in skeletal musculature during exercise conditioning and age-related sarcopenia.^93^ Anatomically distinct myogenic niches harbor dormant muscle-specific stem cells (MuSCs) that maintain lifelong regenerative potential. Myodegenerative events elicit a coordinated immunobiological cascade involving innate and adaptive immune responses. PGE2 can directly target MuSCs through the Ptger4 receptor, promoting their proliferation.^94^

### Metal ions

As an integral component of the musculoskeletal system, bone serves dual functions in providing structural support and facilitating movement while also acting as a reservoir for essential minerals, such as calcium, phosphorus, and various trace elements. The primary composition of bone is calcium phosphate, specifically in the form of hydroxyapatite.^95^ Calcium ions (Ca^2+^) function as ionic messengers, engaging in a multitude of cellular processes, including exocytosis, apoptosis, and cellular motility.^96^ During skeletal regeneration, Ca^2+^ serves as a divalent cation mediator coordinating three critical phases: (1) platelet activation/aggregation through Ca^2+^-dependent integrin signaling, (2) hemostatic plug assembly via fibrinogen crosslinking, and (3) osteogenic mineralization through hydroxyapatite nucleation. These ionic mechanisms synergistically drive the multiphase regenerative cascade. Additionally, trace metallic ions within bone tissue serve as co-factors for enzymatic reactions and are vital for bone metabolism and remodeling. For instance, strontium ions (Sr^2+^) are known to enhance immune suppression and promote osteogenesis.^97^ Excessive iron levels exert harmful impacts on mesenchymal stem cells (MSCs), disrupting their functionality, differentiation capacity, hematopoietic support roles, epigenetic regulation, and the signaling pathways associated with reactive oxygen species.^98^ Magnesium ions (Mg^2+^) establish an immunomodulatory microenvironment while potentiating osteogenic commitment in MSCs through bone morphogenetic protein-2 (BMP-2) signal transduction activation.^99^

### Other factors

Macrophage colony-stimulating factor (M-CSF), secreted by osteoblasts, is a crucial hematopoietic growth factor that plays a significant role in the improvement of both the proliferation and differentiation of osteoclast progenitor cells.^100^ Furthermore, M-CSF facilitates the transformation of osteoclast precursors into fully mature osteoclasts, which possess bone resorptive capabilities. This process is enhanced by the interaction of M-CSF with RANKL, which is expressed on the surface of osteoblasts. The binding of RANKL to RANK, located on the cell surface of osteoclast precursors, initiates critical signaling pathways that lead to bone resorption.

The osteoblast lineage-derived Semaphorin 3A (Sema3A) is pivotal in the processes of postnatal bone remodeling.^101^ Sema3A functions as an osteoprotective agent by attenuating osteoclastic bone resorption while augmenting osteoblastic bone formation. The administration of Sema3A in murine models confers protective effects against bone loss induced by ovariectomy.^102^ Additionally, Sema3A facilitates bone formation during fracture healing and distraction osteogenesis.^103,104^ Notably, Sema3A derived from osteocytes and osteoblasts enhances osteocyte viability and preserves the homeostasis of long bones and lumbar vertebrae under estrogen signaling regulation.^105^

Sphingosine-1-phosphate (S1P) functions as a coupling factor between osteoclasts and osteoblasts, facilitating osteoblast proliferation and enhancing bone formation.^106^ It can be released from osteoclasts into the basic multicellular unit through the transporter SPNS2. S1P operates as both an intracellular signaling molecule and an extracellular messenger. Elevations in sphingosine kinase 1 expression and activity, which leads to increased S1P levels, have been noted during the process of osteoclastic differentiation. Furthermore, osteoclast precursor chemotaxis to osseous remodeling sites is modulated by S1P gradient dynamics and cognate receptor expression profiles. S1P orchestrates bidirectional regulation of bone turnover processes, exerting pleiotropic effects on skeletal mass accrual and biomechanical competence, while demonstrating clinical utility as a diagnostic biomarker for osteoporosis-related fracture risk stratification. Pharmacological modulation of S1P receptor signaling networks or S1P lyase activity represents a novel therapeutic paradigm for osteoporosis management.

Ephrin B2 (Efnb2) plays a crucial role in maintaining bone homeostasis.^107^ It is expressed in various cell types, including mesenchymal stem cells, chondrocytes, osteoblasts, osteocytes, and osteoclasts. During the process of bone development, Efnb2 promoted the differentiation of osteoblasts and facilitated intramembranous bone formation in calvarial organ cultures. Furthermore, Efnb2 enhanced endochondral bone formation by enhancing chondrocyte differentiation and mediating interactions between chondrocytes and osteoclasts. The ablation of Efnb2 in osteoblasts diminished the anabolic effects of parathyroid hormone (PTH) on bone in vivo and negatively impacted the activation, migration, and cartilage matrix formation of human mesenchymal stem cells in vitro. Additionally, treating cells derived from osteoarthritic joints with Efnb2 reduced their production of catabolic factors, indicating the potential therapeutic applications of Efnb2.

Semaphorin 4D (Sema4D) has been identified as a significant suppressor of the bone anabolic process.^108^ It is synthesized by osteoclasts and plays a crucial role in the negative regulation of osteoblast activation that is mediated by insulin-like growth factor 1 (IGF-1). This inhibitory effect occurs through the binding of Sema4D to plexin B1, which acts as its receptor. In a noteworthy contrast, the administration of anti-Sema4D monoclonal antibodies has been shown to effectively restore the diminished bone content in osteoporosis-induced ovariectomized mice. This finding underscores the potential translational significance of targeting Sema4D as a therapeutic approach in the treatment of bone lytic disorders.

Thus, BDFs play a crucial role in mediating the musculoskeletal system’s function. Recently, Liang et al.^4^ discovered 375 osteokines by employing a holistic, integrative, and multi-omics systems biology methodology, detailing their cellular origins. Notably, several previously unrecognized osteokines were identified, and their functional roles warrant further investigation. Grasping the intricate interactions among these variables is vital for clarifying the mechanisms that govern bone metabolism and their significance for comprehensive health and disease management.

## BDFs and metabolic regulation

**Fig. 3:**
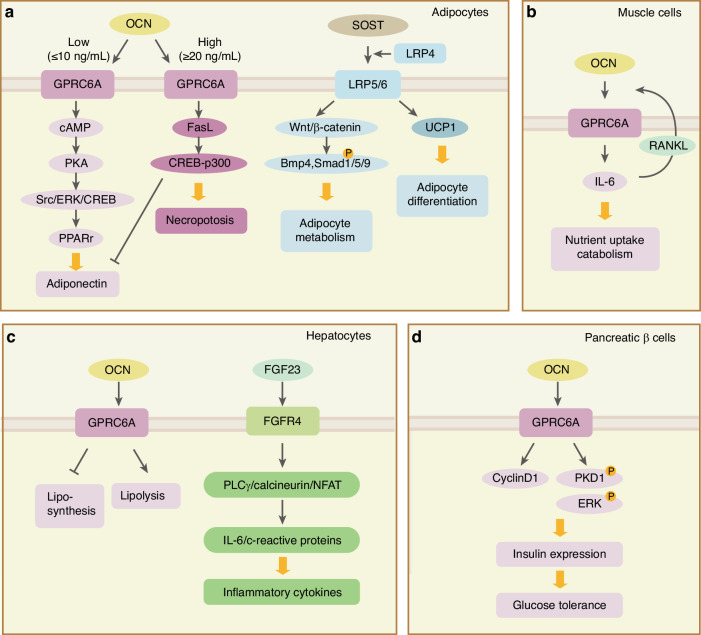
Bone-derived factors mediate metabolism. Bone-derived soluble proteins, including OCN, FGF23, sclerostin, DKK1, OPG, LCN2, IL-11, Irisin and SLIT2 are demonstrated with essential regulatory functions in the metabolic process of remote tissues such as fat, muscle, liver and pancreas. **a** In adipocytes from fat tissue, low dose of OCN induces GPRC6A receptor activation, which leads to the induction of adiponectin expression by cAMP-PAK-Src/ERK/CREB-PPARγ axis; High dose of OCN upregulates FasL and its downstream CREB-p300, resulting in enhanced necroptosis and reduced adiponectin formation. Moreover, sclerostin interacts with Lrp5 and Lrp6 by the co-receptor LRP4 and downstream effectors and finally influence adipocyte differentiation and metabolism. **b** In muscle, OCN signaling in myofibers enhanced the exercise-induced release of IL-6 from muscles, leading to nutrient uptake and catabolism. Enhanced release of IL-6 form muscles can activate the RANKL expression and further OCN express in osteoblasts. Together, this OCN-IL6-RANKL positive feedback loop forms a muscle-bone-muscle endocrine axis increasing muscle function during exercise. **c** In hepatocytes, OCN inhibits lipo-synthesis and induces lipolysis through GPRC6A receptors; FGF23 stimulates the expression of inflammatory cytokines including IL-6 and c-reactive proteins through FGFR4 receptor and downstream PLCγ/calcineurin/NFAT signaling. **d** In pancreatic β cells, OCN enhances insulin expression and glucose tolerance through CPRC6A receptor and downstream PKD1 and ERK, which maintains the β-cell function in insulin secretion and glucose metabolism

BDFs not only govern bone metabolism but also contribute to the preservation of physiological homeostasis. As one of the most important organs that are largely involved in physical activities, bone also contributes to the regulation of glucose and lipid metabolism occurring in the remote soft tissues. Bone-derived soluble proteins, OCN, Fibroblast growth factor 23 (FGF23), sclerostin, Dickkopf-related protein 1 (DKK1), OPG, Lipocalin 2 (LCN2), IL-11, Irisin, glucocorticoid signaling and recently reported secreted glycoproteins SLIT2 are demonstrated with essential regulatory functions in the metabolic process of liver, fat and muscles (Fig. 3). The interplay between bone and glucose/lipid metabolism is a complex and intriguing area of research. Recent studies have highlighted the role of bone-derived factors in mediating lipid and glucose metabolism.

### OCN

During matrix mineralization, OCN is used as a marker for mature osteoblasts and early osteocytes.^109^ A large number of experimental mouse studies demonstrate that bone-derived OCN proteins actively regulate glucose metabolism, insulin signaling, and cellular differentiation in the pancreas, adipocytes, livers, and muscles (Table 1). In mice, OCN deficiency (*Ocn*^*−/−*^) mice are fat and glucose intolerant; ex vivo studies showed that OCN can stimulate cyclinD1 and insulin expression in β-cells.^110^ Moreover, ucOCN administration improved glucose tolerance and increased insulin secretion and sensitivity, resulting in mice displaying decreased fat mass and becoming lean.^110^ This result directly showed that bone-derived OCN can increase insulin secretion of β-cells in the pancreas. A follow-up study proved that GPRC family C group 6 member A (GPRC6A) is the OCN-sensing receptor in β-cells by introducing GPRC6A deficient (*Gprc6a*^*−/−*^) mice.^111^ Furthermore, specifically inactivated the *Gprc6a* gene in the β-cell lineage (*Gprc6a*^*Pdx1*^ mice) demonstrated the GPRC6A in receiving OCN signal and responding in a cyclin-D1 dependent manner.^112^ Together, these results illustrate an OCN/GPRC6A signaling pathway controlling β-cell proliferation in insulin regulation in pancreas mass accrual.

**Table 1:** Bone-derived factors mediate glucose/lipid metabolism

Protein	Cell Type	Receptor	Model	Function	Ref.
Ocn	osteoblast			Bone formation	^110–112^
	adipocytes	Gprc6a	*Ocn*^*−/−*^ mice; in vitro 3T3-L1 adipocytes	Low-dose ucOCN enhances adiponectin expression by cAMP/PAK/Src/ERK/CREB/PPARγ pathway; high-dose ucOCN triggers necroptosis through Fas signaling	^110,113,114^
	white adipose tissue	Gprc6a	ucOCN treated obese mice; 3T3-L1 adipocytes	Upregulate glucose transporter 4 protein and its mRNA (Slc2a4); Increase insulin signaling; downregulate inflammation	^115^
	brown adipose tissue	Gprc6a	Brown adipocytes (mice DE-2-3 cells and BAT-ADSCs)	Ocn-mediated activation of Gprc6a, futher activate Tcf7 and its downstream target proteins during thermogenesis	^116^
	liver	Gprc6a	liver-specific GPRC6A knockout (*GPRC6A*^*LKO*^) mice	UcOCN inhibits lipid synthesis and promots lipolysis.	^117^
	muscles	Gprc6a	*Ocn*^*−/−*^, *GPCR6A*^*−/−*^ mice	Exogenous osteocalcin treatmentprevent age-related muscle loss in mice	^119^
	myofibers	Gprc6a	*Ocn*^*Osb−/−*^, *Gprc6a*^*Mck-/−*^ mice, *Ocn*^*−/−*^, *Il6*^*Hsa–/–*^, *Il6r*^*osb–/–*^ mice	Favor uptake and catabolism of glucose and fatty acids; muscle-derived IL-6 sends signal in osteoblasts and osteocalst and futher enhance the rellease of ucOCN in circulation	^118,120^
Fgf23			*Fgf23*^*−/−*^ mice	Growth retardation, hypoglycemic, increased peripheral insulin sensitivity, improved subcutaneous glucose tolerance.	^12,125^
	hepatocytes	Fgfr4	Cultured hepatocytes, *Fgfr4*^*−/−*^ mice	Activate Fgfr4, further stimulate PLCγ/calcineurin/NFAT signaling, leading to increased expression and secretion of inflammatory cytokines	^126^
Sost	white adipose tissue	Wnt signaling	*Sost*^*−/−*^ mice	Active BMP signaling, enhance adipocyte differentiation and metabolism, increase fat mass	^130^
	adipocytes		*Sost*^*−/−*^ and *Sost*^*Dmp1*^	Block the differentiation of PDGFRα^+^ adipoprogenitors to mature adipocytes	^131^
	bone marrow adipose tissue	Wnt signaling	*Sost*^*−/−*^	Regulates the fate determination of bone marrow-adipocyte progenitors by inhibiting the Wnt signaling	^129^
	adipocytes	Lrp4/5/6	*Lrp4*^*Adipoq*^ and *Lrp4*^*Ocn*^	Lrp4^AdipoQ^ displayed increased glucose and insulin tolerance and reduced serum fatty acids; *Lrp4*^*Ocn*^ displayed accumulate body fat and develop impairments in glucose tolerance and insulin sensitivity	^134^
	beige adipocytes	Wnt signaling	*Gsα*^*Dmp1*^	Sclerostin inhibits Wnt signal and activated Ucp1, resulting in beige adippogenesis and less fat mass	^132^
Lcn2	neurons	Mc4r	*Lcn2*^*osb−/−*^ mice	Osteoblast-derived Lcn2 maintains glucose homeostasis by inhibiting food intake.	^152^
	beta cells (pancreas)	NA	*Col1α1-Lcn2Tg*, lcn2 siRNA, db/db	Increase β-cell numbers and functions, improve insulin sensitivity, counteract metabolic dysregulation in obesity.	^154^
IL-11	adipocytes	NA	*IL-11*^*−/−*^, *Ocn-Cre*; *IL-11*^*fl/fl*^, *Adipoq-Cre*; *IL-11*^*fl/fl*^	Enhance osteogenesis and bone formation in bone, suppress adipogenesis in adipocyte tissue and increase energy supply	^156^
Irisin	adpipocytes	NA	Voluntary wheel-running exercise model	Enhance osteogenesis in bone and thermogenesis in adipose tissues	^157^
Glucocorticoid signaling	adipocytes	NA	*Col1α1-Hsd11b2Tg*	Control of leptin resistance, obesity and insulin resistance during aging.	^158^
SLIT2-C	adpipocytes	NA	*Shn3*^*−/−*^*, Osx-Cre; Shn3*^*fl/fl*^*, Adipoq-Cre; Shn3*^*fl/fl*^*, Osx-Cre; Slit2*^*fl/fl*^*, Adipoq-Cre; Slit2*^*fl/fl*^	Control osteogenesis and glucose metabolism.	^160^

*Ocn* osteocalcin, *Gprc6a* G protein-coupled receptor, family C, group 6, *ucOCN* undercarboxylated osteocalcin, *Fgf23* fibroblast growth factor 23, *Sost* sclerostin, *Lrp4* lipoprotein receptor-related protein-4, *Lcn2* lipocalin 2, SLIT2, *Shn3* Schnurri-3, *NA* not available

Besides the pancreas, *Ocn*^−/−^ mice exhibit glucose intolerance, hyperlipidemia, and increased adiposity.^110^ Ocn can improve glucose tolerance, and mice had increased lean and decreased fat mass and triglyceride levels.^110^ In cultured 3T3-L1 adipocytes, ucOCN can enhance adiponectin (an insulin-sensitizing adipokine) expression and reduce insulin resistance.^113^ The detailed molecular mechanism involved in unOCN-induced GPRC6A receptor activation, which accumulated cAMP accumulation and further led to PAK activation and its downstream Src/ERK/CREB activation.^113^ As a result, activated CREB up-regulated expression of peroxisome proliferator-activated receptor γ (PPARγ), which in turn led to the induction of adiponectin expression.^113^ Interestingly, all of these protective mechanisms that lead to adiponectin expression were only reported in ucOCN low-dose (≤10 ng/ml) treatment, high-dose (≥20 ng/ml) ucOCN induced upregulation of FasL at the plasma membrane of 3T3-L1 adipocytes, which further activated CREB-p300 and induced necroptosis.^114^ Moreover, published studies indicate that OCN regulates white adipose tissue (WAT) and brown adipose tissue (BAT) through different mechanisms. In an experiment with unOCN-treated obese mice, OCN improved insulin resistance by decreasing inflammation and increasing insulin signaling and the expression of Slc2a4/GLUT4 in WAT.^115^ Another study demonstrated that Gprc6a in OCN-mediated activation up-regulated T cell factor 7 (TCF7) expression and its downstream target expression in BAT.^116^ Activated TCF7 also enhanced Gprc6a and uncoupling protein 1 (UCP1) expression, a core molecule for uncoupling respiration from ATP synthesis in the mitochondria and core molecular for BAT thermogenesis.^116^

In addition to adipocytes, OCN also actively participates in lipid metabolism in livers and glucose metabolism in muscles. In livers, ucOCN alleviates NAFLD in high-fat diet-fed wild-type mice through the GPRC6A by inhibiting lipid synthesis and promoting lipolysis.^117^ In muscular tissue, OCN signaling enhances glucose and fatty acid uptake and metabolism during physical activity by promoting the utilization of these substrates.^118^ In young mice, exogenous Ocn is able to enhance their exercise capacity.^118^ In aged mice, exogenous Ocn treatment can prevent age-related muscle loss.^119^ In vivo data also showed that Ocn signaling in myofibers enhanced the exercise-induced release of interleukin-6 (IL-6) from muscles.^118^ IL-6, as a myokine that promotes adaptation to exercise and increases exercise capacity by promoting nutrient uptake and catabolism in myofibers, also activated the RANKL expression in osteoblasts through IL-6 receptor in these cells.^120^ These expressed RANKL signals further activated the osteoclast differentiation in bone and contributed to the release of the bioactive form of OCN, unOCN, from the acidic osteoclast microenvironment to the general circulation.^120^ As a result, this feedback loop forms a muscle-bone-muscle endocrine axis that is necessary to increase muscle function during exercise in rodents and humans.^120^

### Fibroblast growth factor 23

FGF23 is a hormone derived from bone and bone marrow that plays a critical role in maintaining phosphate homeostasis.^10,121^ The primary function of FGF23 is to lower serum phosphate levels by reducing renal reabsorption of phosphate and the synthesis of 1,25-dihydroxyvitamin D (1,25(OH)_2_D_3_).^122^ In both humans and mice, FGF23 contains 251 amino acids with a molecular weight of 32 kD. There are two major post-translation regulations of FGF23, including phosphorylation and O-glycosylation. On one side, FGF23 is phosphorylated by the extracellular kinase family member 20 (CFAM20C) at the site of S180 and further recognized and cleaved by the subtilisin-like proprotein convertase FURIN.^123^ After this proteolysis process, the phosphorylated FGF23 is cut into an inactive N-terminal fragment and a blocking C-terminal fragment.^123^ On the other side, O-glycosylation of FGF23 through polypeptide N-acetylgalactosaminyltransferase 3 (GALNT3) at the side of T178 can prevent phosphorylation and proteolysis of FGF23, leading to secretion of intact and bio-active full-length of FGF23 in the circulation.^123^

Reported studies showed that FGF23 is tightly involved in glucose metabolism and insulin sensitivity in different organs (Table 1). Deletion of the *Fgf23* gene (*Fgf23*^*−/−*^) in mice showed severe growth retardation with a short life span and significant bone defect.^124^ Importantly, *Fgf23*^*−/−*^ mice also displayed distinct hypoglycemic with profoundly increased peripheral insulin sensitivity and improved subcutaneous glucose tolerance.^125^ In cultured primary mouse hepatocytes, Fgf23 can directly stimulate the expression of inflammatory cytokines, including IL-6 and C-reactive protein, in these cells.^126^ Moreover, this Fgf23-induced hepatic secretion of inflammatory cytokines was regulated by the Fgf4 receptors in hepatocytes.^126^ The molecular pathway involved in this process is the activation of Fgfr4 by Fgf23, which further enhanced its downstream PLCγ/calcineurin/NFAT signaling.^126^

### Sclerostin

Besides the local functions in regulating bone remodeling, sclerostin is proposed to be involved in the regulation of glucose metabolism in other tissues (Table 1). In healthy human adults, clinical examination found that the serum level of sclerostin is positively correlated with age and fat mass.^127^ Moreover, the serum level of sclerostin increased in prediabetes patients, which is correlated with insulin resistance in their skeletal muscle, liver, and adipose tissue.^128^

Similarly, sclerostin also regulates the metabolism in mouse adipose tissues. Sclerostin directly induced the bone marrow pre-adipocytes into adipocytes by inhibiting the Wnt signaling.^129^ Deletion of the *Sost* gene in mice (*Sost*^−/−^) led to a dramatic increase in bone volume and also a reduction in fat mass and adipose tissue accumulation, which was also associated with increased insulin insensitivity.^130^ Overproduction of sclerostin with adeno-associated virus resulted in the opposite metabolic phenotype due to adipocyte hypertrophy.^130^ These results could result from the activation of BMP signals upon Sost activation in white adipose tissue.^130^ From lineage tracing experiments, *Sost*^*−/−*^ mice showed a clear decrease of PDGFRα^+^ adipoprogenitor cell differentiation toward mature adipocytes, concomitant with β-catenin activation in the Wnt signaling pathway. This molecular interplay culminates in adipose tissue mass reduction and enhanced systemic glucose homeostasis.^131^ In another study conducted with beige adipocytes, deletion of the stimulatory subunit of G-proteins, Gsα, in mature osteoblasts and osteocytes (*Gs*α^*Dmp1*^) resulted in enhanced sclerostin secretion from osteocytes, which further inhibited the Wnt/β-catenin pathway, activated the brown-adipocyte-like uncoupling-protein 1 (UCP1), and lead to dramatic increase in beige adipogenesis and decreased body fat.^132^ These opposite effects on fat mass could be due to the effects of Gsα in multiple hormonal pathways for adipocyte regulation.^133^

To understand the different regulations of circulating sclerostin on bone and adipose tissue, mice strains with conditional knockout of LRP4, a co-receptor for sclerostin’s interaction with the Lrp5 and Lrp6 in Wnt signaling, were generated,^134^ i.e., osteoblast-specific LRP4 deletion (*Lrp4*^*AdipoQ*^) and adipocyte-specific LRP4 deletion (*Lrp4*^*Ocn*^). The reported study showed that *Lrp4*^*Ocn*^ mice exhibited a high bone mass phenotype as expected, dramatic increases in serum sclerostin, and impairments in glucose tolerance and insulin sensitivity with a significant accumulation of body fat.^134^ Interestingly, *Lrp4*^*AdipoQ*^ mice displayed increased glucose and insulin tolerance and reduced serum fatty acids, which resembled the effect of sclerostin deficiency on whole-body metabolism.^134^ These results suggest that LRP4 is required in both osteoblasts and adipocytes for normal sclerostin endocrine functions. However, sclerostin has distinct physiological functions in different cellular backgrounds.^134^ Importantly, recently published results suggested that a subgroup of adipose lineage cells in bone marrow also expressed a relative level of sclerostin and contributed to the bone formation process.^65^ Conditional deletion of *the Sost* gene in adiponectin-expressing cells (*Sost*^*Adipoq*^) resulted in normal serum levels of sclerostin, leptin, and adiponectin in mice, but these mice displayed a significantly increased bone mass by promoting osteoblast differentiation in bone microenvironment from 3 months onwards.^65^ Moreover, another study showed that exercise training in wild-type male mice induced a reduction of subcutaneous white adipose tissue (scWAT) and scWAT adipocyte cell size, which might be due to increased Lrp4 protein and mitochondrial content after exercise training in adipocytes.^135^

### Dickkopf1

DKK1 was first identified as a regulator of embryonic head development in *Xenopus laevis*.^136^ This, containing 266 amino acids and secreted protein with 28.7 kDa molecular weight, is reported in subsequent studies to have essential functions in regulating embryogenesis, organogenesis, and homeostasis.^137^ Under homeostatic conditions, DKK1 is constitutively produced by bone-forming osteoblasts and mechanosensory osteocytes within the osseous niche through paracrine/autocrine secretory mechanisms.^138^ However, elevated DKK1 expression was detected in various cell types under different disease conditions, including bone disease, diabetes, cancer, rheumatism, and Alzheimer’s disease (AD).^139^ DKK1 functions as a canonical antagonist of Wnt signaling by competitively binding to LRP5/6 co-receptors, thereby disrupting ligand-receptor complex formation through induced co-receptor internalization from the cell surface.^140^ Similar to the mechanism of sclerostin, DKK1 also inhibits the activation of canonical Wnt signals in targeted cells.

Reported studies showed that bone-derived DKK1 had important functions in bone mass regulation under different metabolic conditions. Mice with loss of DKK1 in mature osteoblasts and osteocytes (*Dkk1*^*Dmp1*^) were completely protected from Type 1 diabetes mellitus (T1DM)-induced cortical bone loss.^141^ Moreover, conditional deletion of the Dkk1 gene in osteolineage cells by Osx-Cre (*Dkk1*^*Osx*^) and Dmp1-Cre (*Dkk1*^*Dmp1*^) showed protective effects in glucocorticoid-induced bone loss.^142^ In addition, loss of DKK1 in osteoprogenitor cells mitigated high-fat diet (HFD)-induced cortical porosity development and pathological marrow adipocyte expansion, while paradoxically inducing trabecular volume depletion characteristic of uncoupled metabolic osteopathy.^143^ These observations together suggest an important function of bone-derived DKK1 in sensing and responding to external lipid levels and a promising therapeutic target, especially for bone diseases.

In liver tissue, liver-originated DKK1 directly participates in lipid metabolism. In cultured hepatocytes, DKK1 regulates hepatic lipid metabolism in two ways^137^: one is through activation of the ERK-PPARγ-CD36 axis to enhance the uptake of fatty acids in hepatocytes; another way is by activating JNK signaling to increase insulin resistance in these cells. As a result, DKK1 in livers regulates hepatic lipid metabolism.^137^ Besides hepatocytes, DKK1 also regulates the differentiation of adipose-derived stem cells (ACSs). In vitro, anti-DKK1 treatment, but not anti-Sost, promoted ACS osteogenic differentiation by enhancing the canonical Wnt signaling pathway in ACSs.^144^

### Lipocalin 2

LCN2 is a circulatory protein with 198 amino acids in humans (200 amino acids in mice). LCN2 was first reported to have high expression by fat cells and is considered an adipokine that promotes insulin resistance.^145^ Following up studies demonstrate that LCN2 is present in a large variety of cells and tissues, including bone cells, hepatocytes, lung, adipose tissue, macrophages, prostate, renal cells, and cancer cells.^146,147^ Because of its wide expression, LCN2 has different functions, such as anti-bacterial, anti-inflammatory, anti-aging, and protection against cell and tissue stress, depending on the tissue environment.^146,148^ Importantly, it has been demonstrated that osteoblasts are the primary source of LCN2, whose expression in osteoblasts is at least 10-fold higher than that in white adipocytes.^149^

In bone, LCN2 is expressed throughout osteoblast differentiation and acts as an inhibitor for bone formation.^150^ The study showed that over-expression of LCN2 (LCN2-Tg) in bone resulted in thinner cortical bone and reduced trabecular numbers, which is due to the increased osteoclast progenitors and reduced osteoblast differentiation followed by LCN2-induced RANKL and IL-6 expression in these Tg mice.^150^ However, global deletion of *Lcn2* in mice (*Lcn2*^*−/−*^) also resulted in reduced bone formation and increased glucose tolerance.^151^ This could be because the external LCN2 down-regulated the important bone metabolism protein, glucose transporter protein type 1, in osteoblasts.^151^

Osteoblastic LCN2 coordinates systemic glycemic control through endocrine crosstalk mechanisms.^152^ The secreted LCN2 can cross the blood-brain barrier,^153^ bind and activate the melanocortin four receptor (MC4R) of neurons from the hypothalamus, and inhibit food intake.^152^ Interestingly, serum LCN2 levels were reported to have a positive correlation with insulin levels and β-cell functions, whereas it also correlated with body mass index and insulin resistance in the same individuals from a clinical examination and also in mouse studies.^154^ Furthermore, in vivo studies showed that overexpressing LCN2 (Col1α1-Lcn2Tg) in osteoblasts decreases food intake and fat mass as well as improves glucose metabolism.^154^ On the contrary, decreased circulating LCN2 by systemic delivery of *Lcn2* siRNA resulted in worsened hyperphagia, fat mass, body weight gain, and glucose metabolism in both db/db and diet-induced obese mice.^154^ Together, these data demonstrate a protective mechanism of circulating LCN2 in the maintenance of β-cell function and counteract metabolic dysregulation.^152,154^

### Other factors

In addition to the BDFs mentioned above, several new factors that originated from bone but function in remote tissues during metabolic regulation were recently reported. For example, interleukin (IL)-11 is a type of cytokine first secreted from helper T cells, which has been reported to have multiple tissue originations and functions with a broader spectrum of biological regulations. Published data showed that IL-11 is mainly expressed in bone and is up-regulated by mechanical loading (Table 1).^155^ These stimulated upregulation of IL-11 proteins from osteoblasts have two distinct functions: in bone, IL-11 enhances osteogenesis locally by inhibiting sclerostin, resulting in increased bone mass; in the adipose tissue, IL-11 suppresses the expression of Wnt inhibitor, Dkk1 and 2, ending up with the suppression of adipogenesis and increased energy supply for exercise.^156^ Moreover, Irisin, known as one important myokine, is up-regulated at both mRNA and protein levels in murine bone tissues in a two-week voluntary wheel-running exercise model, which further leads to an increase in circulating Irisin levels in mice and finally contributes to osteogenesis and uncoupling protein 1 (UCP1)-mediated thermogenesis in adipose tissues^157^ (Table 1). Furthermore, another group generated a transgenic mouse strain with deleted glucocorticoid signaling in osteoblasts and osteocytes by overexpressing 11β-hydroxy-steroid dehydrogenase type 1 type 2 (11 β-HSD2), an important enzyme that determines glucocorticoid concentration, under the *Col1α1* gene (Table 1). These Col1α1-Lcn2Tg showed reduced insulin resistance and leptin resistance and remained lean during aging.^158^ The key to managing body weight in aged Col1α1-Lcn2Tg mice may lie in the fact that their adipose tissue retains leptin sensitivity in terms of oxygen consumption rate.^158^

Currently, a recent study indicated that glycoprotein SLIT2 is another important BDF secreted from osteoblasts and functions in fat tissue (Table 1). Schnurri-3 (SHN3, also known as Hivep3) is an adapter protein that functions intrinsically within osteoblasts to inhibit their bone-forming capabilities.^159^ The absence of Shn3 not only enhanced osteoblast activity but also alleviated obesity-related metabolic disorders.^160^ *Shn3*^*−/−*^ mice exhibited resistance to diet-induced obesity and an improvement in the browning of white adipose tissue. Conditional deletion of Shn3 specifically in osteoblasts or AAV-mediated silencing of Shn3 in bone tissues replicated the lean phenotype and enhances glucose metabolism. Sequencing data and proteomics data further indicated that the C-terminal fragment of SLIT2 (SLIT2-C), predominantly released by osteoblasts, was a BDF regulated by Shn3, which played a critical role in mediating white adipose tissue browning. Thus, a bone**–**fat signaling pathway mediated by SHN3 regulates the production of SLIT2-C in osteoblasts, presenting a promising therapeutic target for the treatment of both osteoporosis and metabolic syndrome.

Several other proteins, such as OPN and BMPs, were first reported to be expressed in bone tissue and involved in bone homeostasis, which was demonstrated with wide expression in other tissues during the regulation of metabolism. OPN was initially considered a bone-derived extracellular matrix (ECM) glycoprotein and plays a critical role in bone remodeling. Extensive data showed that OPN is abundantly expressed in the liver, brain, kidney, and placenta of humans and mice, which has been proven with participation in several disease conditions, including inflammation, liver fibrosis, and non-alcoholic fatty liver disease.^161^ Epididymal white adipose tissue-resident macrophages constitute a cellular source of OPN, which undergoes endocrine transport to the osseous niche.^162^ This adipokine mediates bone ECM remodeling through paracrine activation of osteoclast-mediated resorptive activity. Another important secreted factor is BMP, which is also originally identified as a bone-derived osteoinductive component and participates in a wide array of processes during various organ formation and maintenance.^163^ For example, BMP4 exerts multifunctional protective effects in hepatocytes through YAP/TAZ pathway modulation, demonstrating senescence suppression, steatosis attenuation, inflammation resolution, and fibrogenesis inhibition.^164^ In HFD mouse liver, BMP9 can improve glucose and lipid metabolism, decrease inflammatory responses, and reshape chromatin accessibility, resulting in alleviating NAFLD phenotype in mice.^165^

## Central nervous system

**Fig. 4:**
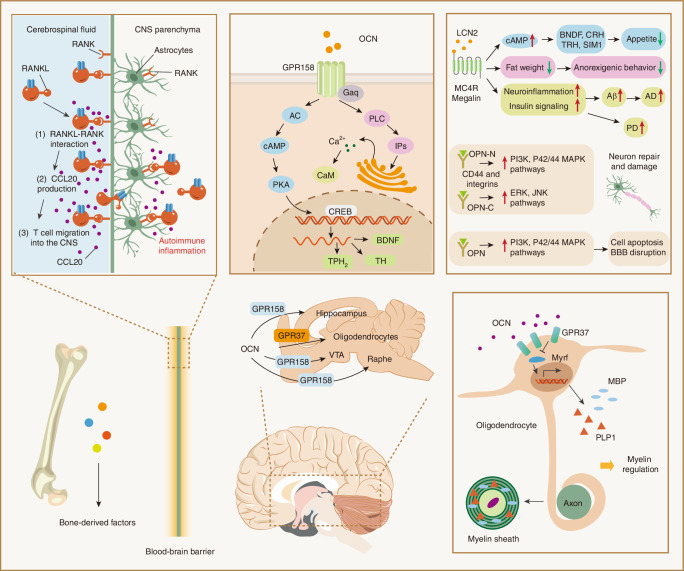
The schematic illustration elucidates the role of BDFs within the central nervous system. BDFs, which encompass Osteocalcin (Ocn), RANKL, LCN-2, and Osteopontin (OPN), possess the capacity to traverse the blood-brain barrier (BBB). Within the cerebral architecture, BDFs engage with various receptors, including Gpr37, Gpr158, MC4R, and Megalin, distributed across distinct brain regions, thereby modulating the synthesis of a range of neurotransmitters. Collectively, these interactions enable BDFs to influence processes such as spatial learning, memory formation, appetite regulation, myelin maintenance, neuronal repair, and neuroinflammatory responses

The CNS functions as a master regulatory organ composed of interconnected neural circuits essential for orchestrating systemic homeostasis.^166^ Emerging translational evidence has established bidirectional regulatory crosstalk between osseous and neural systems, wherein skeletal homeostasis critically modulates neurodevelopmental trajectories and cognitive performance.^167^ Pathological bone remodeling correlates with neurological dysfunction progression, with recent studies delineating neurotropic regulatory capacity of BDFs including OCN, LCN2 and OPN in neurodegenerative pathologies (Fig. 4).

### OCN

Peripherally, OCN signals through the G protein-coupled receptor GPRC6A to execute systemic endocrine regulation, modulating insulin secretion, androgenic steroid biosynthesis, and exercise-induced myocyte adaptation.^168^ Centrally, blood-brain barrier (BBB)-permeable OCN suppresses γ-aminobutyric acid (GABA)ergic neurotransmission while enhancing catecholamine/serotonin synthesis via selective engagement with neuronal subpopulations in hippocampal, brainstem, and mesencephalic nuclei (Table 2).^169^ GPR158, the first identified CNS-resident OCN receptor, is constitutively expressed in primary cortical domains, the retro-splenial area, and the ventral tegmental area, governing sensorimotor integration and auditory processing.^170^ Moreover, OCN has been shown to be a bioactive molecule in oligodendrocytes by activating Gpr37, a substrate of the E3 ubiquitin ligase parkin.^171^

**Table 2:** Bone-derived factors in the CNS

Protein	Cell type	Receptor	Model	Function	Ref.
Ocn	Anterior hippocampus, cortex	Gpr158	*Gpr158*^−/−^ mice behavioral and electrophysiological assays	Facilitate the maintenance and adequate restoration of cognitive function	^170^
	Different brain regions and cells, particularly enriched in mature oligodendrocytes	Gpr37	*Ocn*^−/−^ mice	Modulation of oligodendrocyte differentiation and myelination via the downstream transcription factor Myrf	^171^
	Substantia nigra and Striatum	AKT-GSK3β signaling	6-hydroxydopamine (6-OHDA)-induced PD rat model	Enhance the therapeutic efficacy in addressing behavioral dysfunction and mitigate the reduction of tyrosine hydroxylase within the nigrostriatal pathway, while also inhibiting the proliferation of astrocytes and microglia.	^174^
Lcn2	Paraventricular nucleus and ventromedial neurons of the hypothalamus	Mc4r	*Col1α1-Cre* and *Bglap-Cre*, *Lcn2*^*fl/fl*^	(1) Modulation of neuronal cell apoptosis, migration patterns, and morphological characteristics. (2) Inhibition of appetite	^152^
	The inner surface of cerebral endothelial cells, neutrophils, and astrocytes	NA	*Lcn2*^*−/−*^ mice, Transient Middle Cerebral Artery Occlusion model	The deletion of Lcn2 or the application of Lcn2 monoclonal antibodies notably mitigates cerebral damage following a stroke	^179^
		NA	*Lcn2*^*−/−*^ mice	Lcn2 deficiency diminishes cerebral edema, neuronal loss, neutrophilic infiltration, and impairments in behavior.	^180^
	Hippocampus	NA	*Lcn2*^*−/−*^ mice	Facilitate stress-related alterations in spinal structure and performance to modulate neuronal excitability and anxiety levels.	^181^
	Substantia nigra	Lcn2r	A neurotoxin model of PD	Protect the nigrostriatal dopaminergic system	^178^
Opn	Cortical and hippocampal regions	NA	Glatiramer acetate-immunized ADtg mice, *Opn*^*−/−*^ mice	Enhanced absorption of Aβ fibrils along with related scavenger receptors and anti-inflammatory responses	^184^
	The perforation side of brain tissue	NA	Aneurysmal subarachnoid hemorrhage rat model	Mitigate initial cerebral damage and suppress neuronal cell death.	^185^
	Microglia and macrophages within the infarct core and in peri-infarct regions	Cxcr4	Mice photothrombosis surgery for ischemia	Stimulate neurogenesis from neural stem cells in vitro, while also enhancing stem cell survival, proliferation, and migration	^186^
	Bronchial epithelium cells	NA	Particulate matter-induced acute airway inflammation mouse model	Potentiate inflammatory cytokines via the ERK and JNK pathways	^189^
Rankl	Neurons and astrocytes in the preoptic area and the Medial Septal nucleus	Rank	*Nestin-Cre* and *GFAP-Cre*; *Rankl*^*fl/fl*^	The activation of brain regions associated with thermoregulation leads to fever induction through the COX2-PGE2/EP3R signaling pathway.	^192^
	T cells	Rank	*Tnfsf11*^*flox/D*^ mice; *Lck-Cre*	The suppression of Rankl inhibited the progression of encephalomyelitis while leaving the peripheral immune response unchanged.	^194^
	Activated microglia and macrophages	Rank	*Opg*^*−/−*^ mice or recombinant RANKL-treated mice, Transient middle cerebral artery occlusion model	Mitigate inflammation via the Toll-like receptor signaling cascade in microglial cells.	^195^

*Ocn* osteocalcin, *Gpr* G protein-coupled receptor, *Lcn2* lipocalin 2, *Opn* osteopontin, *Rankl* receptor activator of nuclear factor kappa B ligand, *NA* not available

OCN was reported to have essential regulatory functions in myelin thickness and myelin sheath severity. The levels of myelin proteins MBP, MAG, and PLP1 in the CNS white matter were significantly elevated in the corpus callosum, spinal cord, and cerebellum of OCN-deficient mice compared to their wild-type littermates at postnatal day 30 (P30).^171^ There was a substantial increase in the quantity of CC1^+^ mature oligodendrocytes in OCN-deficient mice, indicating the premature differentiation of oligodendrocytes. The intracerebroventricular infusion of synthesized mouse OCN led to a significant increase in OCN levels in the corpus callosum and spinal cord regions, along with a decrease in BrdU^+^/CC1^+^ differentiated OLs and CC1^+^ cells. Conversely, the activity of OCN was blocked by infusing OCN antibodies into wild-type mice, resulting in a significant up-regulation of PLP1 and MBP in the corpus callosum region. These findings suggest that OCN plays a crucial role in the regulation of myelination in adults. Behavioral experiments revealed that adult mice lacking OCN displayed a substantial increase in anxiety-like behavior and had a significant deficit in memory and learning.^170^ Administration of OCN to OCN-deficient mice reduced anxiety-like behavior and improved their memory and learning capabilities.^172^ Furthermore, maternal OCN was found to be necessary for fetal brain development, regulation of neurogenesis, prevention of neuronal apoptosis, and optimal memory and spatial abilities in adult offspring.^173^ In rat Parkinson’s disease models, OCN levels were decreased in the cerebrospinal fluid, but injection of bone-derived OCN significantly elevated dopaminergic cell survival via AKT/GSK3β pathway.^174^

OCN demonstrates neuroprotective efficacy in cerebral ischemia models, mediated through proline hydroxylase 1 suppression to enhance neuronal viability.^175^ Mechanistically, OCN reprograms glucose flux toward the pentose phosphate pathway, thereby attenuating pyroptotic cell death. Clinical correlational analysis reveals acute ischemic stroke patients with elevated serum OCN concentrations exhibit improved neurological recovery trajectories compared to counterparts with persistent neurological deficit severity. Complementary murine studies utilizing *Runx2* haploinsufficient models delineate OCN’s role in mitigating age-associated cognitive impairment and anxiety phenotypes.^170^ These findings propose a dual therapeutic axis: (1) perinatal nutritional optimization to prevent developmental osteocalcin deficiency-linked neuropsychiatric sequelae; (2) OCN administration for reversing neurocognitive aging, independent of developmental programming.^42^

### LCN2

Lipocalin 2 (LCN2) is a member of lipocalin superfamily that is secreted into the circulatory system.^146^ It has been identified to bind cell surface receptors, such as Megalin, 24p3R and MC4R.^176^ LCN2 is also called neutrophil gelatinase-associated lipocalin (NGAL) because it was first discovered in the gelatinase subcellular compartment of human neutrophils.^177^ LCN2 was initially believed to be an adipokine mainly expressed in adipose tissues and induced by dexamethasone (Dex) and TNF-α.^145^ In fact, it is primarily expressed in the liver, adipose tissue, and bone (Table 2). Reported data showed that osteoblastic LCN2 exhibits an order-of-magnitude elevation in biosynthetic output compared to adipose-derived stromal cell populations.^152^ This cell-type-specific expression gradient underscores LCN2’s preferential skeletal origin in physiological contexts.

Neuroinflammation serves as a significant factor in numerous conditions of the CNS, such as AD, Parkinson’s disease (PD), stroke, multiple sclerosis (MS), spinal cord injury, and LPS-induced sepsis. LCN2 stimulates the proinflammatory activation of glial cells and, in certain circumstances, may enhance the infiltration of neutrophils and macrophages into the brain. Osteoblast-specific LCN2 knockout murine models developed hypophagic phenotypes accompanied by elevated gonadal adiposity and total adipocyte mass. Clinical biomarker profiling revealed divergent LCN2 dynamics in neurodegenerative cohorts: cerebrospinal fluid (CSF) LCN2 depletion concurrent with plasma elevation in mild cognitive impairment (MCI) and AD patients.^9^ Plasma LCN2 levels demonstrated inverse correlation with Clinical Dementia Rating (CDR) scores and positive association with Mini-Mental State Examination (MMSE) metrics, establishing its prognostic utility for tracking MCI-to-AD conversion trajectories. Lipocalin-2 (LCN2) orchestrates AD pathogenesis through tripartite pathomechanisms: exacerbating neuroinflammatory cascades via microglial activation, dysregulating insulin/IGF-1 signaling axis in neuronal populations, and amplifying Aβ plaque-associated gliosis through astrocyte reactivity potentiation. In an animal study, researchers observed an increase in LCN expression in the substantia nigra after injecting 6-hydroxydopamine into the medial forebrain bundle, indicating a potential association between elevated LCN2 levels and PD pathogenesis. Furthermore, they discovered that LCN2 ablation improved PD symptoms in mice, suggesting a possible pathogenic mechanism involving disruption of the nigrostriatal dopaminergic projections, abnormal locomotor behaviors, neurotoxic iron accumulation, and neuroinflammation.^178^ Therefore, developing regulatory methods for LCN2 or inhibitors of LCN2-induced neurotoxicity and neuroinflammation may be beneficial for treating PD. Clinical biomarker profiling revealed circulating LCN2 concentrations demonstrate significant elevation during the hyperacute phase of acute ischemic stroke, which might induce post-stroke infections and cardiovascular mortality.^177^ In a mice model of ischemic stroke, LCN2 in serum was strikingly increased after stroke but decreased post-ischemia,^179^ which could be considered as an early blood biomarker for stroke. Subsequent mechanistic investigations in *Lcn2*-null murine stroke models demonstrated attenuated manifestations of BBB compromise, neurological dysfunction severity, cerebral infarct volume, and neutrophilic infiltration. Experimental evidence further delineated LCN2-mediated neuroinflammatory amplification via tripartite mechanisms: (1) neutrophil recruitment dynamics, (2) microglial/astrocytic gliosis potentiation, and (3) transcriptional upregulation of inflammatory mediators (cytokines/chemokines). *Lcn2* knockout (heterozygous or homozygous) mice presented decreased brain swelling, neutrophil infiltration, microglia activation, and neuronal death after being subjected to an intracerebral hemorrhage model.^180^ LCN2 also plays a role in stress-responsive neuroadaptation through hippocampal signaling modulation. Mucha et al.^181^ found that stress-induced LCN2 upregulation in murine hippocampal circuits precipitated dendritic spine attrition and neuronal hyperexcitability, manifesting as anxiety-like behavioral phenotypes. Collectively, these findings establish LCN2 as a pleiotropic mediator of neuroinflammatory pathogenesis across neurological disorders, positioning pharmacological modulation of LCN2 expression or ligand-receptor interactions as a therapeutic target for CNS conditions.

### OPN

OPN, a secretory matricellular protein first identified in osseous tissue, belongs to the small integrin-binding ligand N-linked glycoprotein (SIBLING) family with pan-tissue expression patterns (renal parenchyma, epithelial interfaces, striated muscle, mammary glands, CNS)^182^ (Table 2). As a key ECM mineralization modulator, OPN exerts pleiotropic regulatory effects in neurotrauma, cerebrovascular ischemia, and neurodegenerative pathologies.^182,183^ Basal CNS OPN expression localizes predominantly to olfactory bulb mitral cells, cerebellar Purkinje neurons, and pontomedullary nuclei, exhibiting rostral-caudal expression gradients.

OPN demonstrates a dual regulatory paradigm in CNS pathophysiology: (1) Neuroprotective functions in acute injury via inflammatory cascade modulation, apoptotic suppression, BBB integrity maintenance, and neural progenitor chemotaxis; (2) Pathological exacerbation through chronic gliosis potentiation. Mechanistically, OPN serves both structural (ECM scaffold) and signaling roles (integrin/RGD domain interactions), coordinating post-injury matrix remodeling and neural repair mechanisms.^184^ Research using a rat model of intravascular perforation has shown that the endogenous OPN and autophagy-related proteins are elevated, suggesting that OPN promotes the function of autophagy to inhibit early brain damage and neuronal apoptosis.^185^ In cerebral hemorrhage models, OPN upregulation induces microglial and macrophage activation, driving neuroblast migration and proliferation to enhance neural regeneration and functional recovery.^186^ OPN deficiency disrupts macrophage chemotaxis and proinflammatory cytokine synthesis,^187^ while its presence stimulates myelinogenesis and remyelination, critical for repairing aberrant neuronal circuits in AD.^188^

Paradoxically, OPN exhibits proinflammatory exacerbation through JNK/ERK-mediated cytokine overexpression.^189^ It further aggravates BBB integrity, neurogenesis, and angiogenesis post-injury. In brain tissues of AD rats, OPN is mainly expressed and increased in inflammatory plaques, which is positively correlated with age and Aβ deposition.^190^ These results suggest that the increase in OPN may indicate severe neurodegeneration and pathological changes. The conflicting findings may be explained by the formation of different OPN fragments after protease cleavage, which can bind to distinct receptors (CD44 and integrins) and activate other signaling pathways and cell responses.^191^

### RANKL

Emerging evidence demonstrates constitutive expression of RANKL and its cognate receptor RANK within the CNS, where they exhibit novel capabilities (Table 2). In recent years, researchers have found that RANKL/RANK is involved in female thermoregulation and the central fever response in inflammation.^192^ Central injections of RANKL in mice and rats triggered severe fever. RANKL signaling engages thermoregulatory brain nuclei via the COX2-PGE2/EP3R signaling axis to mediate febrile responses. RANK-deficient female murine models displayed elevated basal thermoregulatory setpoints, revealing sex-specific RANKL/RANK-mediated hypothalamic temperature modulation. Human RANK Arg170Gly homozygous mutation carriers with autosomal recessive osteopetrosis.^193^ During severe pulmonary infections requiring hospitalization, these people demonstrated attenuated febrile responses compared to immunocompetent pediatric cohorts showing characteristic pneumonia-induced pyrexia with antibiotic-responsive resolution. This suggests that RANKL/RANK also controls human body temperature. This intriguing discovery raises questions about the broader implications of RANK mutations beyond bone health. The impaired fever response observed in these patients suggests a potential role of RANKL/RANK in regulating the body’s immune and inflammatory responses. In autoimmune pathogenesis, T cell-derived RANKL orchestrates astrocytic C-C motif chemokine ligand 20 (CCL20) biosynthesis, establishing chemotactic gradients that govern CNS T cell infiltration.^194^ T cell-specific RANKL knockout murine models demonstrated complete experimental autoimmune encephalomyelitis resistance through impaired neuroantigen-specific T cell CNS trafficking. Therapeutic RANKL inhibition via monoclonal antibodies achieved CNS-specific immunomodulation, effectively suppressing encephalomyelitic progression while preserving peripheral adaptive immunity, suggesting that RANKL could be a potential therapeutic target for treating autoimmune diseases in the CNS. In autoimmune diseases, the regulation of C-C type chemokine ligand 20 (CCL20) production by astrocytes is influenced by RANKL on T cells, consequently impacting T cell localization in the CNS. The findings open up new possibilities for targeted therapeutic interventions aimed at modulating T-cell trafficking without compromising peripheral immune responses. This could pave the way for more effective treatment strategies for autoimmune diseases with CNS involvement. In stroke, the RANKL/RANK signaling pathway protects neurons and relieves nerve damage.^195^ OPG, RANKL, and RANK mRNA are increased in the acute stage of ischemic stroke and are expressed in activated microglia and macrophages. On the one hand, enhanced RANKL/RANK signaling contributes to the reduction of infarct volume and brain edema, leading to reduced post-ischemic inflammation. On the other hand, reduced RANKL/RANK signaling increases infarct volume. Increased OPG could be a causal factor in reducing RANKL/RANK signaling and increasing post-ischemic inflammation.

As BDFs have been confirmed to participate in interorgan communication, it can be inferred that bone likely actively regulates physiological processes in the CNS. These factors are believed to be crucial in controlling gene expression related to the differentiation and communication of various cell types responsible for maintaining CNS homeostasis. Given the complex interactions between bone and brain, further research is necessary to fully understand the content and potential modulatory roles of BDFs in regulating CNS functions. Therefore, variations in the expression levels of these factors may serve as a promising diagnostic or prognostic tool for detecting early-stage CNS disorders.

## BDFs and the circulatory system

**Fig. 5:**
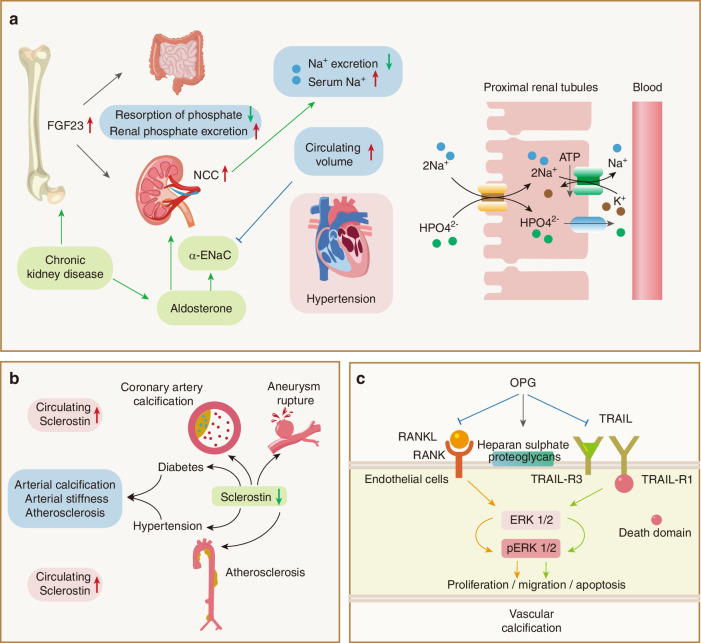
Proposed model of BDF-mediated axis of bone-circulating system. **a** Elevated levels of circulating FGF23 enhance the expression and activity of NCC in the distal renal tubules, subsequently resulting in renal sodium retention, volume expansion, hypertension and hypertrophy. **b** Sclerostin inhibition results in the development of abdominal aortic aneurysms and atherosclerosis. However, high serum concentrations of sclerostin correlate with increased atherosclerosis, severe vascular calcification, and elevated arterial stiffness. **c** The role OPG in the pathophysiology of vascular calcification. OPG interacts with RANKL, thereby obstructing the RANKL/RANK signaling pathway. Additionally, OPG demonstrates direct agonistic properties on vascular endothelial cells through its heparin-binding domain. Furthermore, OPG acts as a competitive inhibitor of TRAIL by hindering TRAIL-death receptor interactions

The circulatory system is intimately linked to the skeleton through a range of BDFs that mediate crosstalk between the two systems. These BDFs, including platelet-derived growth factor-BB (PDGF-BB), OPG, RANKL, FGF23, and sclerostin, play critical roles in bone remodeling and development. Moreover, their roles in the regulation of various processes in the cardiovascular system, such as angiogenesis and cardiomyocyte proliferation, are also becoming increasingly recognized (Fig. 5).

PDGF-BB, a disulfide-bonded homodimeric protein comprising two identical PDGF-B subunits, is a constituent of the PDGF signaling family. Preosteoclasts (osteoclast precursors) exhibit significantly elevated secretory capacity for PDGF-BB.^196^ PDGF-BB is one of the most active cytokines in the PDGF family. Skeleton-secreted PDGF-BB binds to PDGF receptors and mediates cardiovascular diseases through various signaling pathways.^197,198^ First, PDGF-BB can promote excessive proliferation of pulmonary artery smooth muscle cells via the PI3K/AKT signaling pathway, causing pulmonary arterial hypertension.^199^ Second, PDGF-BB regulates the proliferation, invasion, and migration of vascular smooth muscle cells (VSMCs) through miR-212-5p/YWHAZ and miR-149-5p/NRP2 axis, leading to atherosclerosis.^200,201^ In addition, PDGF-BB can also induce dedifferentiation in VSMCs by enhancing TXNIP level via sponging miR-513a-5p.^202^

There is more and more research data confirming that OPG is involved in cardiovascular disease processes and is a prognostic indicator of cardiovascular diseases, including atherosclerotic lesions, ischemic heart failure, and acute myocardial infarction.^203^ OPG exerts the above biological effects mainly by three signaling pathways in the endothelial cells. First, OPG antagonizes RANKL signaling through competitive inhibition of RANKL-RANK binding, blocking osteoclastogenesis.^204^ Second, OPG exerts direct agonistic effects on vascular endothelium via its heparin-binding motif, mediating angiogenesis regulation.^205^ Third, OPG competitively inhibits TNF-related apoptosis-inducing ligand (TRAIL) by preventing TRAIL-death receptor interactions, thereby suppressing apoptotic cascades.^206^ Initially, increased OPG concentration may reflect the vascular wall’s response to damaging stimuli; however, sustained high levels can activate detrimental OPG pathways, resulting in vascular injury and atherosclerosis.^203^ The OPG/TRAIL signaling axis presents a promising therapeutic target in cardiovascular diseases. The complexities of OPG/RANKL/TRAIL interactions on vascular cells remain incompletely elucidated, and further investigation is needed to mitigate adverse OPG effects while enhancing TRAIL efficacy in the vascular system.

Elevated serum levels of FGF23 are correlated with a heightened mortality risk in individuals suffering from acute heart failure^207^ or chronic heart failure.^208^ FGF23 can regulate cardiovascular function through various signaling pathways.^209^ One of the most important signaling pathways is the FGF23/FGFR1c/Klotho pathway. FGF23 binds to its cognate receptor FGFR1c and Klotho on cardiomyocytes, leading to the activation of ERK1/2 and AKT signaling pathways. These signaling cascades promote cardiomyocyte proliferation and survival, as well as angiogenesis.^210^ Elevated circulating FGF23 levels were also tightly associated with an increased risk of heart failure with chronic kidney disease (CKD) in a large multicenter prospective cohort study.^211^ Mechanistically, FGF23 attenuates phosphate reabsorption in both renal and intestinal systems. FGF23 enhances the expression and activity of the distal renal tubular sodium-chloride cotransporter (NCC), promoting sodium retention, subsequent volume expansion, hypertension, and cardiac hypertrophy. In a counter-regulatory response, hypernatremia and elevated blood volume suppress aldosterone secretion by the adrenal glands, which downregulates renal α-epithelial sodium channel (α-ENaC) expression.

Sclerostin plays a role in the regulation of cardiovascular function.^212^ Besides bone cells, sclerostin is also expressed in endothelial cells, smooth muscle cells, and cardiomyocytes within the cardiovascular system. It binds to its receptor LRP5/6 to activation of ERK1/2, JNK, and p38 MAPK kinases.^63^ These kinases phosphorylate various substrates, leading to changes in gene expression, protein synthesis, and cell behavior.^213^ One of the genes regulated by SOST is vascular endothelial growth factor (VEGF), a key angiogenic factor. Sclerostin suppresses VEGF expression in endothelial cells, leading to reduced angiogenesis and blood flow.^214^ However, the role of sclerostin varies in different physiological contexts.^215^ Several investigations indicated that serum sclerostin levels exhibited a positive correlation with arterial calcification, arterial stiffness, and the severity of atherosclerosis. Conversely, aortic sclerostin expression was shown to be downregulated in a murine model of abdominal aortic aneurysm, while transgenic overexpression or exogenous administration of sclerostin was demonstrated to mitigate the development of abdominal aortic aneurysm and atherosclerosis. Furthermore, the inhibition of sclerostin was associated with an increased incidence of hypertension and diabetes, thereby heightening the risk of cardiovascular events.

The mechanisms underlying these regulatory roles involve complex signaling networks that involve multiple cell types and tissues, such as endothelial cells, smooth muscle cells, and cardiomyocytes, leading to changes in gene expression, protein synthesis, and cell behavior (Table 3). Understanding these regulatory mechanisms is crucial for developing effective strategies to manipulate cardiovascular function and disease risk.

**Table 3:** Bone-derived factors in the circulatory system

Protein	Cell Type	Receptor	Model	Function	Ref.
PDGF-BB	Pulmonary artery smooth muscle cells	PDGF receptors	Monocrotaline induced Pulmonary arterial hypertension in rats	Pulmonary artery smooth muscle cell proliferation and inflammation	^199^
	Vascular smooth muscle cells		serum from atherosclerosis patient	vascular smooth muscle cell injury	^200^
Opg	Macrophage, smooth muscle cells	Rankl-Rank	*ApoE*^*−/−*^ *Opg*^*−/−*^ mice	Slow down lesion progression and vascular calcification	^204^
Fgf23	Cardiomyocytes	Fgfr and Klotho	5/6-resection of renal tissue in rats	Promote cardiomyocyte proliferation and survival	^209^
Sost	Endothelial cells, smooth muscle cells, and cardiomyocytes	Lrp5/6		Anti-Sost antibody increases myocardial infarction risk	^63,215^

*PDGF-BB* platelet-derived growth factor-BB, *Opg* osteoprotegerin, *Rankl* receptor activator of nuclear factor kappa B ligand, *Ocn* osteocalcin, *Fgf23* fibroblast growth factor 23, *Sost* sclerostin, *Lrp* lipoprotein receptor-related protein, *NA* not available

## BDFs and the reproductive system

The reproductive system and the skeleton are interconnected through a myriad of signaling molecules and hormones. BDFs, such as OCN and FGF23, play a pivotal role in this communication (Table 4).^6,216^

**Table 4:** Bone-derived factors in the reproductive system

Protein	Cell Type	Receptor	Model	Function	Ref.
Ocn	Leydig cells of the testes gland	Gprc6a	*Gprc6a*^*−/−*^ mice	Modulate the expression of enzymes necessary for testosterone synthesis in a manner dependent on Creb, thereby enhancing the survival of germ cells.	^111^
	Leydig cells	Gprc6a	*Ocn*^*–/–*^ and *Gprc6a*^*–/–*^ male mice	Promote testosterone biosynthesis in the mouse testis	^281^
Fgf23	Oocytes	Fgfr1	Mice 18.5 days postcoitum to 1 day postpartum	Mitigate oocyte apoptosis in the perinatal murine ovary through the activation of the p38 MAPK signaling cascade.	^221^
Opn	Cumulus cells and oocytes	Integrins and CD44	In vitro experiments	Enhance cumulus cell expansion and oocyte fertilization	^225,226^

*Ocn* osteocalcin, *Gprc6a* G protein-coupled receptor, family C, group 6, *Fgf23* fibroblast growth factor 23, *Opn* osteopontin

OCN is a hormone produced by osteoblasts that play a crucial role in male fertility. Research has demonstrated that the undercarboxylated variant of OCN can boost fertility in genetically modified mice. This enhancement is attributed to the increased production of testosterone and a higher sperm count resulting from the presence of this OCN form.^217^ OCN exerts a direct effect on testosterone synthesis by interacting with the G protein-coupled receptor class C group 6 member A (GPRC6A) located on Leydig cells. This interaction subsequently impacts the testosterone receptors present in Sertoli cells. When the undercarboxylated form of OCN binds to its receptors on Leydig cells, it initiates the production of cAMP. The rise in cAMP levels then activates the CREB1. Activation of CREB1 prompts the expression of key genes involved in testosterone biosynthesis, such as the steroidogenic acute regulatory protein (StAR) and the cytochrome P450 family 11 (CYP11). These proteins are crucial for the synthesis of testosterone.^218,219^

The role of FGF23 in the reproductive system is more complex. FGF23 is an osteocyte-derived hormone that acts on the regulation of Phos, Ca and vitamin D metabolism.^6^ In males, FGF23 has been shown to regulate testosterone production directly through Leydig cells.^220^ However, its effects on female reproductive function are less clear, with some studies showing anabolic effects on uterine tissue while others suggest catabolic effects on ovarian follicles.^221,222^ The mechanisms behind these effects are likely due to the differential expression of FGF23 receptors in male and female reproductive tissues. The regulatory functions of OCN and FGF23 in the reproductive system are not confined to their direct impacts on hormone production; they also engage with a spectrum of other regulatory elements. These interactions are part of a complex endocrine network that modulates various physiological processes, including reproduction. For instance, OCN has been shown to modulate the effects of Gonadotropin-releasing hormone (GnRH) on pituitary gonadotropes, amplifying the release of Luteinizing hormone (LH) and Follicle-stimulating hormone (FSH).^223,224^ Similarly, FGF23 has been demonstrated to interact with other signaling molecules, such as BMP and Wnt, altering their downstream effects on reproductive tissues.^6^

In addition to OCN and FGF23, OPN, DKK1 and RANKL also play essential roles in the reproductive system. OPN is a secreted glycoprotein that regulates ovulation, implantation, and parturition. OPN promotes ovulation by binding to integrins and CD44 receptors on cumulus cells and oocytes, enhancing cumulus cell expansion and oocyte fertilization.^225,226^ OPN affects prostaglandin E2 production in cumulus cells, which is required for ovulation.^227^ DKK1, a Wnt signaling inhibitor, has been shown to regulate female fertility through its effects on oocyte development and ovulation.^228,229^ DKK1 modulates female fertility by inhibiting Wnt signaling in oocytes, which is essential for oocyte development and meiotic division. DKK1 modulates progesterone production in luteal cells, which is necessary for maintaining pregnancy.^228,229^ RANKL, a member of the tumor necrosis factor superfamily, has also been shown to affect gonadal steroidogenesis by interacting with its receptor RANK on Leydig cells. RANKL signaling modulates Leydig cell function, affecting testosterone production and potentially impacting male fertility.^230,231^

In summary, BDFs play a crucial role in the crosstalk between the skeleton and the reproductive system. These factors regulate gonadal steroidogenesis, fertility, and gene expression in reproductive organs through various mechanisms. Understanding the regulatory effects and mechanisms of BDFs on the reproductive system provides valuable insights into potential novel therapeutic targets for the treatment of reproductive disorders.

## BDFs and renal functions

**Fig. 6:**
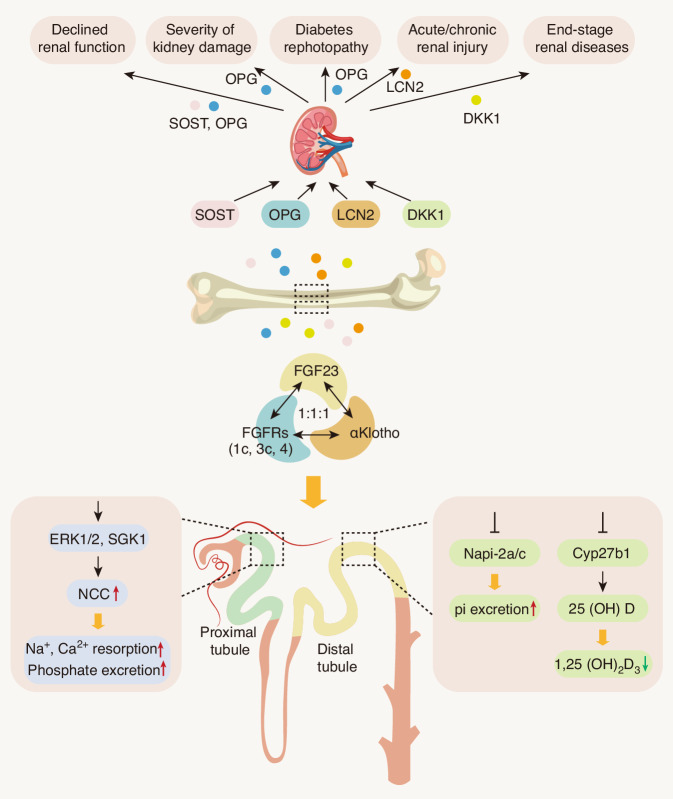
Bone-derived factors regulate renal functions. Clinical data and animal studies demonstrate that multiple bone-derived factors, including FGF23, sclerostin, OPG, LCN2 and DKK1, are tightly associated with normal kidney functions, kidney damage and different stages of renal diseases. Among all of these factors, the molecular mechanisms of FGF23 in the regulation of kidney functions are most illustrated. FGF23 binds to αKlotho and FGFRs together and from a 1:1:1 ternary complex, regulating renal functions at the different parts of renal tubes. In distal tubules, FGF23 contributes to the Ca^2+^ and Na^+^ reabsorption and phosphate excretion by enhancing NaCl co-transporter NCC through ERK1/2 and SGK1 pathways. In proximal tubules, FGF23 interacts with FGFR1 and downregulates the expression of membrane NaPi-2a and NaPi-2c, resulting in enhanced phosphate (pi) excretion. Moreover, FGF23 reduces the mRNA level of Cyp27b1 and suppress its downstream production of 25(OH)D, leading to the inhibition of 1,25(OH)2D3 synthesis

FGF23, sclerostin, DKK1, LCN2 and OPG have been reported with tight association to renal functions predominantly by their endocrine regulations. Even though these factors originate from bone, they are largely involved in the progress of CKD, directly contribute to kidney development, and regulate the phosphate reabsorption (Fig. 6).

### FGF23

As one of the superfamilies of fibroblast growth factors (FGFs), FGF23 can bind to FGF receptors, including FGFR1c, FGFR3c, FGFR4 and transmembrane protein αKlotho.^232,233^ αKlotho acts as a scaffold protein that is required for the high-affinity bind of FGF23 to their cognate FGF receptors (FGFRs).^234^ Reported data showed that αKlotho binds to FGFRs and FGF23 together and form a 1:1:1 ternary complex.^235^ Because αKlotho shows tissue-specific expression in human samples with high expression in kidney and renal artery,^236^ this may explain the mainly endocrine functions of FGF23 in kidney.^232^

The *Fgf23*^*−/−*^ mice displayed significantly high serum phosphate with increased renal phosphate reabsorption.^124^ These mice also had an elevation in serum 1,25(OH)2D due to the enhanced expression of renal 25-hydroxyvitamin D-1α-hydroxylase (1α-OHase) from 10 days of age.^124^ In the kidney, FGF23 is well documented in the regulation of renal functions in two parts: one is phosphate excretion, and the other is 1,25(OH)_2_D_3_ formation. For the renal phosphate regulation, studies showed that administration of recombinant FGF23 caused a decrease of serum phosphate level in mice, and implantation of FGF23-expressing cells into nude mice lead to hypophosphatemia and decreased 1,25(OH)_2_D_3_ formation.^237^ Moreover, overexpressing human FGF23 in mice caused renal phosphate wasting, which is accompanied by reduced expression of type II sodium-phosphate cotransporter NaPi-2a in the epithelial cells of the renal proximal tubules.^238^ For the second regulation, FGF23 can suppress the synthesis and induce the degradation of 1,25(OH)_2_D_3_, the hormonally active form of vitamin D. Animal studies showed that FGF23 reduced the mRNA level of 25-hydroxyvitamin D-1α-hydroxylase (*Cyp27b1*) in renal tissues, which is the key enzyme for the production of active 1,25(OH)_2_D_3_ from 25(OH)D.^239^ Moreover, FGF23 also increased the mRNA level for 25-hydroxyvitamin D-24-hydroxylase (*Cyp24α1*) in renal tissues, which inactivates 1,25(OH)_2_D_3_.^239^

Detailed cellular and molecular mechanisms of FGF23’s regulating functions at the different parts of renal tubes were illustrated with transgenic animal studies. In distal tubules, FGF23 contributes to the reabsorption of sodium (Na^+^) and calcium (Ca^2+^). In these cells, FGFR1 co-localized with Klotho is the principal mediator of FGF23 effects.^240^ FGF23 enhances the membrane abundance of NCC through extracellular signal-regulated kinase 1/2 (ERK1/2), serum/glucocorticoid-regulated kinase 1 (SGK1), and with-no lysine kinase-4 (WNK4) pathways.^241^ Immunohistochemical profiling localized FGFR3 expression to murine renal proximal tubular epithelia, while targeted genetic deletion of FGFR3 or FGFR4 failed to attenuate FGF23-driven renal pathophysiological responses, indicating compensatory signaling redundancy in FGF23-mediated nephron regulation.^240^ Further studies showed that FGF23 interacts with FGFR1 and downregulates the expression of membrane sodium-phosphate cotransporter NaPi-2a and NaPi-2c.^242^ This process is mediated by FGF23-induced serine phosphorylation of the scaffolding protein Na^+^/H^+^ exchange regulatory co-factor (NHERF)-1 through ERK1/2 and serum/glucocorticoid-regulated kinase-1 (SGK1) signaling.^243^

In short summary, circulating FGF23 mainly originates from bone, and elucidating FGF23’s mechanistic pathways and its renal downstream signaling interactions may provide a foundation for innovative therapeutic development targeting phosphate-regulating disorders.

### Other factors

Several other BDFs were reported to be tightly associated with renal functions. Clinical samples from non-dialysis chronic kidney disease (ND-CKD) patients showed that declined renal function was associated with a significant increase in OPG and sclerostin.^244^ Among them, serum OPG levels are tightly associated with the severity of kidney damage,^245^ diabetic nephropathy,^246^ cardiovascular mortality risk in patients with chronic kidney disease,^247^ and 5-year all-cause mortality in patients with chronic kidney disease.^248^ However, in vivo studies to illustrate the detailed regulation of OPG in renal functions are required. Compared to OPG, several published studies deciphered the participation of Sclerostin in kidney homeostasis. In vivo *Sost*^*−/−*^ mice were detected with the phenotype in the setting of chronic renal failure with severe secondary hyperparathyroidism, in addition to the high bone mass phenotype of *Sost* deficiency.^249^ In another study with juvenile cystic kidney (JCK) mouse, a model of polycystic kidney disease (PKD) that carries a mutation of the Nek8 serine-threonine kinase, increased expression of Wnt antagonists (sclerostin and sFRP4) were reported.^250^ These data suggest that the circulating level of sclerostin is associated with kidney function.

Furthermore, clinical analyses revealed significantly elevated DKK1 concentrations in both serum and renal parenchyma of CKD stage 4-5 patients compared to non-CKD controls.^251^ Moreover, the high serum DKK1 CKD group showed a faster progression to end-stage renal disease (ESRD) than the low serum DKK1 CKD group in this 8-year follow-up study.^251^ Animal studies further suggest that DKK1 could act as a profibrotic mediator to accelerate CKD disease progression to ESRD.^251^ In a high glucose (HG)-induced diabetic nephropathy model, DKK1 was reported with functions in HG-induced destabilization of β-catenin and matrix accumulation in mesangial cells, resulting in microstructure deterioration, renal dysfunction and finally diabetic nephropathy.^252^

In addition, LCN2 has been used as a biomarker for acute and chronic renal injury. An animal study from *Lcn2*^*−/−*^ mice showed a complex renal phenotype including polyuria, glycosuria, proteinuria, and renal cortex vacuolization, suggesting a kidney contribution of LCN2 to their phenotype.^151^ Moreover, genetic ablation of *Lcn2* abolished inflammation-induced Fgf23 upregulation, while exogenous Lcn2 supplementation elevated serum Fgf23 concentrations in both wild-type and CKD murine models.^253^ This process is mediated by the stimulation of Fgf23 transcription of LCN2 via activation of cAMP-mediated signaling in bone cells.^253^ However, since LCN2 is a multi-original and multifunctional secreted protein that is involved in many organs and different disease models,^254^ a detailed regulatory mechanism between bone-derived LCN2 and renal functions needs to be further illustrated.

## BDFs and the immune system

Bone functions as the principal lymphoid organ that houses hematopoietic stem cells (HSCs) and immune progenitor cells. The bone and immune cells coexist within the same microenvironment, where they are influenced by various regulatory factors, including cytokines, chemokines, and receptors, which collectively facilitate the homeostasis of the ‘osteoimmune system’. The emerging discipline of osteoimmunology has shed light on the intricate interactions between the skeletal and immune systems, which are crucial for understanding the pathogenesis of immune-related disorders.

### RANKL

RANKL serves as a pivotal BDF that connects skeletal and immune systems, vital for molecular and cellular processes determinant in secondary lymphoid organ development.^255^ Rankl-deficient mice demonstrate abnormalities in the early differentiation of T and B lymphocytes, lacking all lymph nodes. In addition, RANK and RANKL-deficient animals display defects in Peyer’s patches and cryptopatches, as well as abnormalities of the spleen.

The investigation of the downstream signaling pathways associated with the RANKL-RANK axis in osteoclastogenesis has uncovered numerous shared molecules and signaling mechanisms that link bone and immune cells.^255^ Mice deficient in immunoreceptor tyrosine-based activation motif (ITAM)-containing adapters, specifically the Fc receptor common gamma subunit (FcRγ) and DNAX-activating protein (DAP)12, display pronounced osteopetrosis due to compromised osteoclast differentiation. In osteoclast precursor cells, FcRγ and DAP12 interact with various immunoreceptors, facilitating calcium signaling via phospholipase Cγ activation. RANKL can interact with signals from ITAM-containing immunoglobulin-like receptors, such as TREM2, SIRPβ1, OSCAR, PIR-A, and FcγRIII.^256^ These signaling molecules have been thoroughly characterized within the framework of immune cell development and signaling, encompassing T cells, B cells, and NK cells.

### CXCL12

Within the bone marrow microenvironment, mesenchymal lineage cells constitute specialized niches critical for HSC homeostasis. LepR^+^CXCL12^+^ mesenchymal stem cells form the architectural and functional HSC niche through sustained secretion of stem cell factor (SCF) and CXCL12—crucial mediators of HSC quiescence and niche retention.^257,258^ These multipotent MSCs serve as progenitors for osteoblasts and marrow adipocytes under physiological conditions, while exhibiting fibrogenic potential in pathophysiological contexts.^259^ Osteoclast-derived proteases (MMP9, cathepsin K) proteolytically degrade CXCL12 within the niche, thereby destabilizing HSC anchorage. This mechanistic linkage demonstrates osteoclastic activity functionally regulates HSC egress from medullary compartments.^260^ The ablation of CXCL12 in early osteolineage cells resulted in a reduction of B cell progenitors, while the deletion in osteocytes led to significant metabolic alterations, primary thymic damage, and a decline in the generation of both B and T lymphocytes. In contrast, physical activity-mediated reduction of adipocyte-derived leptin secretion upregulates CXCL12 biosynthesis in LepR^+^ MSCs within the bone marrow microenvironment.^261^ Consequently, this leads to a reduction in inflammatory leukocytes, which contributes to the mitigation of cardiovascular inflammation.

### OCN

Ocn^+^ bone cells (mature osteoblasts) facilitate the generation of T-lineage competent cells and their migration to the thymus through the expression of the Notch ligand DLL4.^262^ The specific ablation of Ocn^+^ cells in vivo resulted in a reduction of DLL4 expression at both the mRNA and protein levels. In addition, the population of T-competent progenitors and the expression of thymus-homing receptors among hematopoietic cells in the bone marrow were reduced. These findings indicated that the decreased expression of DLL4 from Ocn^+^ cells resulted in diminished intracellular Notch signaling in hematopoietic precursors in the bone marrow, thereby establishing a direct connection between skeletal biology and the generation of T cell-mediated adaptive immunity.

### Other factors

Osteoblasts sustain common lymphoid progenitors (CLPs) in the bone marrow through the secretion of interleukin-7 (IL-7).^263^ The ablation of osteoblasts or the inducible deletion of IL-7 specifically in osteoblasts resulted in a lymphopenic phenotype, accompanied by a reduction in CLP numbers, while leaving HSCs unaffected. Pharmacological stimulation of osteoblasts ameliorated sepsis-induced lymphopenia, suggesting that bone cells represent a viable therapeutic target in specific life-threatening immune responses.

Emerging evidence delineates osteoblast-derived extracellular ATP as a critical regulator of bone marrow plasma cell maintenance.^264^ These antibody-secreting lymphocytes depend on P2RX4, a ligand-gated purinergic ion channel, to detect ATP released via osteoblastic pannexin 3 (Panx3) hemichannels. Genetic disruption of *Panx3* or *P2rx4* significantly reduced circulating antibody levels through plasma cell depletion. In vitro models revealed *Panx3*-null osteoblasts exhibit impaired ATP secretion, failing to support plasma cell survival. Pharmacological P2RX4 inhibition (5-BDBD) replicated these effects in vitro, depleted medullary plasma cells in vivo, and suppressed antigen-specific antibody titers with minimal post-treatment recovery, confirming the ATP-P2RX4 axis’s non-redundant role in humoral immunity.

Osteoblastic lineage cells play a crucial role in the maintenance of lymphoid progenitors in the bone marrow, and their dysregulation can result in hematological malignancies. The activation of β-catenin in osteoblasts has been linked to the onset of acute myeloid leukemia^265^ while activating mutations in the protein tyrosine phosphatase SHP2 have also been implicated in leukemia development.^266^ DKK1 derived from osteoblasts were shown to facilitate hematopoietic reconstitution post-myosuppression by directly inhibiting HSC senescence and indirectly promoting epidermal growth factor secretion from bone marrow endothelial cells.^260^ Conversely, other research indicated opposing effects; for instance, subcutaneous administration of Lewis lung carcinoma and B16 melanoma tumor cells resulted in increased DKK1 expression by osteoblasts and osteocytes.^267^ This bone-derived DKK1 subsequently stimulated the expansion of myeloid-derived suppressor cells both in the bone marrow and at tumor sites. Dicer1, an RNase III endonuclease critical for miRNA biogenesis and RNA processing, when deleted in osteoblasts, led to a reduction in *Sbds* gene expression, which is vital for ribosome synthesis. This reduction resulted in compromised osteoblastic differentiation, growth retardation, myelodysplasia, leukemia, and decreased survival.^268^

In summary, osteoimmunology has highlighted the pivotal role of the skeletal system in modulating immune responses, underscoring the intrinsic connection between these two systems. Investigating the mechanisms underlying the interactions among biological systems, as exemplified by BDFs, will be crucial for comprehending the organism as a whole and for advancing innovative therapeutic strategies in the context of inter-organ communication.

## Clinical potential of BDFs

A number of clinical trials are currently underway to explore the therapeutic efficacy of BDFs, particularly focusing on the inhibition of RANKL and Sclerostin in the management of osteoporosis as well as various other bone disorders. Denosumab is a fully human monoclonal antibody that targets RANKL, a crucial player in the pathology of bone loss. The pathway involving RANK and RANKL is recognized as a predominant mechanism underpinning the degradation of bone integrity and loss in conditions like osteoporosis and metastatic bone diseases.^269,270^ By binding to RANKL, denosumab effectively obstructs the formation, function, and longevity of osteoclasts, thus leading to a reduction in bone resorption.^271^ Marketed under the brand names Prolia and Xgeva by Amgen, Denosumab is indicated for a variety of clinical scenarios characterized by elevated bone resorption rates or significant fracture risk. Prolia has received authorization in various regions, including the United States and the European Union (EU), for the management of osteoporosis (both postmenopausal and glucocorticoid-induced) in individuals at elevated fracture risk, as well as for enhancing bone mass in patients at significant fracture risk who are undergoing hormone therapy for breast or prostate carcinoma.^272^ Similarly, Xgeva has been approved in the US, EU, and additional areas for preventing skeletal complications (e.g., fractures, spinal cord compression) in patients with bone metastases from advanced solid tumors, multiple myeloma, as well as unresectable giant cell bone tumors. Denosumab is administered via subcutaneous injection and can reduce bone resorption markers to undetectable levels within a few days. Significant therapeutic effects are observed over the course of months; however, the treatment effects diminish rapidly following cessation of the drug.

In recent years, denosumab, along with its biosimilars, has been the subject of extensive clinical trials globally, encompassing phases I through IV. Beyond osteoporosis, the applicability of RANKL monoclonal antibodies extends to conditions such as fibrous dysplasia, spinal fusion, osteoporotic vertebral compression fractures, and bone metastasis, as indicated in Table 5. Despite a decade’s worth of practical clinical experience with denosumab, there remains an ongoing quest to optimize its application and dosing protocols, as the treatment regimens reported in recent clinical trials exhibit notable variations from one another.

**Table 5:** Bone-derived factors in the clinical trials

Target Protein	Conditions	Drug	Location	Ages and sex	Treatment method	Primary outcome	Phase	Clinical study ID
RANKL	Fibrous dysplasia	Denosumab	USA	Over 18, all	120 mg s.c. Q4W	CTX, PINP	II	NCT03571191
RANKL	Osteopenia, Spine fusion, Denosumab allergy	Denosumab	China	40–85, all	60 mg s.c. plus intravenous placebo Q6M plus calcium supplementation 1200 mg/D and vitamin D 800 IU/D	BMD, P1NP, CTX, VAS score	II	NCT05419050
RANKL	Osteoporotic vertebral compression fracture	Denosumab	China	50–90, all	60 mg s.c. Q6M	BMD, P1NP, CTX, VAS score	II/III	NCT05058443
RANKL	Denosumab allergy, zoledronic acid allergy	Denosumab	China	50–90, all	60 mg s.c. plus intravenous placebo Q6M	BMD, P1NP, CTX, VAS score	IV	NCT05598606
RANKL	Osteoporosis, lumbar fusion	Denosumab	China	Over 50, all	60 mg s.c., on week1 and 26	lumbar fusion rate	IV	NCT05203588
RANKL	Healthy subjects	AVT03	Poland, UK, South Africa	28–55, male	120 mg s.c., single dose	Cmax, AUClast	I	NCT05876949
RANKL	Healthy subjects	AVT03	Australia, New Zealand, South Africa	28–55, male	60 mg s.c., single dose	AUCinf, AUClast, Cmax, CTX-1	I	NCT05126784
RANKL	Healthy subjects	Bmab 1000	USA	28–55, all	60 mg s.c., single dose	AUCinf, AUClast, Cmax	I	NCT05323708
RANKL	Healthy subjects	MW031	China	18–65, male	60 mg s.c., single dose	AUC, Cmax	I	NCT04798313
RANKL	Healthy subjects	LY06006	China	28–65, male	60 mg s.c., single dose	AUCinf, AUClast, Cmax, Tmax	I	NCT04973722
RANKL	Healthy subjects	HS-20090-2	China	18–50, male	60 mg s.c., single dose	AUCinf, Cmax	I	NCT04940845
RANKL	Healthy subjects	CT-P41	Korea	28–55, male	60 mg s.c., single dose	AUCinf, AUClast, Cmax	I	NCT06037395
RANKL	Healthy subjects	HLX14	China	28–65, male	60 mg s.c., single dose	AUCinf, AUClast, Cmax	I	NCT04534582
RANKL	Healthy subjects	CT-P41	Australia	28–55, male	60 mg s.c., single dose	Treatment-emergent adverse events	I	NCT04512872
RANKL	Healthy subjects	MB09	Poland	28–55, male	35 mg s.c., single dose	AUClast, Cmax	I	NCT05299073
RANKL	Healthy subjects	CMAB807	China	18–65, male	60 mg s.c., single dose	AUClast, Cmax	I	NCT03925051
RANKL	Women with osteoporosis	Bmab 1000	UK	55–80, female	60 mg s.c. Q6M	BMD	III	NCT05345691
RANKL	Bone metastases from solid tumors	LY01011	China	18–80, all	120 mg s.c. Q4W	Urinary type I collagen cross-linked N-telopeptides corrected for urine creatinine	III	NCT04859569
RANKL	Bone metastases	MW032	China	over 18, all	120 mg s.c. Q4W	Bone conversion index	III	NCT04812509
RANKL	Postmenopausal osteoporosis	FKS518	Bulgaria, Czechia, Estonia, Georgia, Hungary, Poland	55–85, female	60 mg s.c. every 26 weeks	BMD, P1NP, CTX, Adverse Event	III	NCT04934072
RANKL	Postmenopausal osteoporosis	TVB-009P	USA, Bulgaria, Czechia, Georgia, Hungary, Poland, Russian Federation, Slovakia	60–90, female	60 mg s.c. on various time point	BMD	III	NCT04729621
RANKL	Postmenopausal osteoporosis	CT-P41	Estonia, Latvia, Poland, Ukraine	50–80, female	60 mg s.c., single dose	BMD	III	NCT04757376
RANKL	Postmenopausal osteoporosis	SB16	Poland	55–80, female	60 mg s.c. Q6M	BMD	III	NCT04664959
RANKL	Bone metastases	QL1206	China	18–80, all	120 mg s.c. Q4W	uNTx/uCr	III	NCT04550949
RANKL	Postmenopausal osteoporosis	HLX14	China	60–90, female	60 mg s.c. Q6M	BMD, CTX	III	NCT05352516
RANKL	Postmenopausal osteoporosis	MB09	Bulgaria, Estonia, Georgia, Hungary, Latvia, Mexico, Poland, Serbia	55–80, female	60 mg s.c. Q6M	BMD	III	NCT05338086
RANKL	Postmenopausal osteoporosis	GP2411	USA, Bulgaria, Czechia, Japan, Poland, Spain	55–80, female	60 mg s.c. Q6M	BMD, AUCinf, AUClast, Cmax	III	NCT03974100
RANKL	Postmenopausal osteoporosis	RGB-14-P	USA, Bulgaria, Czechia, Hungary, Italy, Poland, Spain, Ukraine	60–90, female	60 mg s.c. on various time point	BMD, CTX	III	NCT05087030
RANKL	Postmenopausal osteoporosis	ENZ215	Czechia	55–85, female	60 mg s.c., single dose	BMD, CTX	III	NCT05405725
Sclerostin	Osteogenesis imperfecta	Romosozumab	USA, Australia, Germany, Greece, Hungary, Italy, Spain, Turkey	5–17, all	Multiple doses, s.c.	Cmax, Tmax	I	NCT04545554
Sclerostin	Multiple myeloma	Romosozumab	USA	Over 18, all	210 mg s.c., Q4W for 12 months	P1NP, Incidence and Severity of adverse events	I	NCT05775094
Sclerostin	Chronic spinal cord injury and osteoporosis	Romosozumab	USA	Over 18, female	60 mg s.c., monthly	BMD	II	NCT04708886
Sclerostin	Premenopausal idiopathic osteoporosis	Romosozumab	USA	18–45, female	210 mg s.c., once a month for 12 months	Lumbar spine BMD	II	NCT04800367
Sclerostin	Osteoporosis	Romosozumab	China	55–90, female	In a specified sequence	BMD, treatment-emergent adverse events	III	NCT05067335
Sclerostin	Osteogenesis imperfecta	Romosozumab	USA, Australia, Belgium, Canada, France, Germany, Hungary, Japan, Poland, Slovakia, Spain, Switzerland, Turkey, UK	5–17, all	Once a month for 12 months	Number of Clinical fractures/any fractures, lumbar spine BMD	III	NCT05972551
Sclerostin	Osteoporosis	Romosozumab	Denmark	Over 50, female	210 mg/2.34 ml	BMD	IV	NCT06059222
Sclerostin	Spinal cord injuries	Romosozumab	USA	18–55, all	210 mg s.c. every month	BMD	IV	NCT05101018
Sclerostin	Postmenopausal osteoporosis	Romosozumab	India	Over 18, all	210 mg s.c., once a month for 12 months	Treatment-emergent adverse events, clinically significant changes	IV	NCT06079476
Sclerostin	Glucocorticoid-induced osteoporosis	Romosozumab	China	Over 18, all	60 mg s.c. Q6M	BMD	IV	NCT04091243
DKK1	Prostate cancer	DKN-01	USA	18–100, male	Start with 300 mg and be escalated to 600 mg or de-escalated to 150 mg	Number of dose limiting toxicities, number of participants with a best overall response	Ib, IIa	NCT03837353
DKK1	Endometrial cancer	DKN-01	USA	Over 18, all	i.v. over about 30 minutes to 2 hours on Day 1 of each cycle, as well as on Day 15 of Cycle 1.	Incidence of adverse events	II	NCT05761951
DKK1	Colorectal cancer	DKN-01	USA, Germany, Korea,	Over 18, all	i.v. (400 mg) every two weeks with an additional loading dose in the first cycle of treatment	Progression free survival	II	NCT05480306
DKK1	Gastric cancer		USA, Germany, Korea,	Over 18, all	i.v. (300 mg) on Days 1 and 15	Safety and tolerability, Progression free survival	II	NCT04363801

*s.c.* subcutaneous injections, *Q4W* every 4 weeks, *Q6M* every 6 months, *VAS* visual analog scale, *BMD* bone mineral density, *P1NP* procollagen one amino-terminal propeptide, *CTX* C-terminal telopeptide, *AUCinf* area under the concentration infinity, *AUClast* area under the concentration last, *Cmax* maximum serum concentration, *Tmax* time to Cmax, *uNTx/uCr* urinary type I collagen cross-linked peptide adjusted for urinary creatinine, *i.v.* intravenous injections

Sclerostin serves as a critical negative regulator of bone formation, which ultimately leads to a decrease in overall bone formation due to its inhibitory effects on the Wnt signaling pathways that are integral to the process of osteoblastogenesis.^273^ Research has shown that the elimination of sclerostin activity can significantly enhance bone mass across various animal models, as previously discussed in this review. This promising discovery paved the way for a series of clinical trials that revealed remarkable increases in bone mineral density and notable reductions in fracture risk among postmenopausal women treated with romosozumab.^274^ Consequently, the development of monoclonal anti-sclerostin antibodies has emerged as a viable therapeutic strategy for conditions such as osteoporosis, chronic spinal cord injury, and osteogenesis imperfecta (Table 5), with Romosozumab already obtaining marketing authorization in Japan, the United States, and several European countries. However, this breakthrough in improving bone mass through sclerostin inhibition has raised significant concerns about the potential increase in the risk of cardiovascular diseases, including myocardial infarction and stroke. Such risks highlight a previously unrecognized role of sclerostin within cardiovascular tissues. This duality may stem from the expression of sclerostin in various organs beyond the skeletal system, including the kidneys, liver, placenta, fetal skin, heart, brain, thymus, lungs, and pancreas.^275^ Given this multifaceted impact, the targeting of sclerostin has piqued considerable interest, as it may unlock new therapeutic pathways. Moving forward, it is vital that further studies concentrate on the targeted inhibition of sclerostin in bone specifically to develop effective treatments for osteoporosis while minimizing systemic adverse effects.

In addition to sclerostin, DKK1 serves as another secretory antagonist capable of interacting with the Wnt coreceptor, leading to a desensitization of cells to canonical Wnt ligands. DKN-01 is a targeted antibody specifically directed against secreted DKK1, which has been explored both as a monotherapy and in combination with other treatments for a range of malignant tumors, which include endometrial cancer,^276^ colorectal cancer,^277^ gastric cancer,^278^ and prostate cancer^279^ (Table 5). Its efficacy has shown particular promise in patients presenting with tumors that highly express DKK1 or possess identified Wnt mutations.^280^ The complementary mechanisms of action, coupled with minimal adverse effects and emerging biomarker data, position DKN-01 as a notable candidate for combination therapies in patients with advanced malignancies. This mechanism primarily involves an immunomodulatory effect, engaging predominantly with the innate arm of the immune system, which underscores the potential to overcome innate immune resistance and broaden the spectrum of patients who may derive benefits, likely in a manner informed by biomarker selection.

Despite the emergence of groundbreaking and effective therapeutic strategies developed from BDFs, there remains a strong rationale for their utilization in clinical settings. Presently, ongoing research initiatives aim to refine the clinical management approaches for patients by harnessing BDFs in both the treatment and prevention of various diseases. Throughout these endeavors, it is imperative to maintain a steadfast focus on ensuring that safety and efficacy are prioritized as the foremost considerations in any therapeutic application.

## Conclusion and future perspectives

**Fig. 7:**
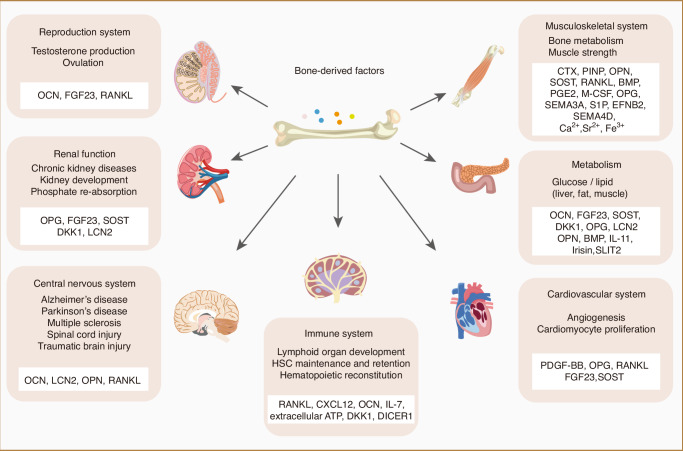
BDFs mediate inter-organ communications in health and disease. Beyond its established function in offering structural integrity, bone is instrumental in preserving the functionality and equilibrium of multiple organs and tissues, such as the muscle, liver, adipose tissue, heart, vasculature, testes, ovaries, kidneys, brain, lymphoid organs, and hematopoietic cells through the secretion of specific bioactive factors

Recent investigations into BDFs have sparked significant interest within the scientific community. The bone is capable of responding to metabolic and energy demands through the secretion of a variety of hormones. The interplay between the skeletal system and other exoskeletal organs, such as the brain, heart, liver, pancreas, kidneys, testes, ovaries, and muscles, underscores the essential endocrine roles of the skeleton. (Fig. 7). These factors exhibit significant hormonal characteristics, facilitating the regulation of organ functions via specific receptors. Their involvement spans various biological systems and is intricately linked to the body’s metabolic processes.

The validation of BDF-mediated interorgan communication is expected to significantly enrich our comprehension of the intricate communication networks that link the skeletal system with other bodily systems, thereby revealing the multifaceted roles that BDFs play in maintaining homeostasis. The systematic exploration of multi-omics methodologies is essential for the identification and characterization of novel BDFs, which may play significant roles in inter-organ communication. Furthermore, it is imperative to conduct thorough in vivo investigations into therapeutic strategies that specifically target these BDFs, as well as their downstream signaling pathways, across a diverse array of transgenic models and disease models. This comprehensive and integrative approach will not only enhance our understanding of the complex interactions involved but also potentially pave the way for the development of more effective treatment options tailored to individual patient needs.

While the roles of BDFs have been extensively validated, it is crucial to acknowledge that the majority of current research has primarily focused on animal models, with legitimate clinical trials being relatively limited. Recently, the paracrine roles of bone-derived EVs have gained significant attention for their involvement in intercellular communication and the transfer of genetic material.^7,14^ However, their physiological functions and the potential of EV-based nanotechnology remain to be elucidated, amidst ongoing debates concerning the study of EVs in controlled laboratory settings. Consequently, there is a pressing need for prospective studies to validate the therapeutic potential of BDFs in clinical settings. It will be essential to elucidate the intricate regulatory networks that govern the relationships between bone and other organs, as this knowledge is critical for the development of targeted and precise therapies aimed at improving patient outcomes in a range of pathological contexts.
